# Public perception of accuracy-fairness trade-offs in algorithmic decisions in the United States

**DOI:** 10.1371/journal.pone.0319861

**Published:** 2025-03-13

**Authors:** Mehdi Mourali, Dallas Novakowski, Ruth Pogacar, Neil Brigden

**Affiliations:** 1 Haskayne School of Business, University of Calgary, Calgary, Alberta, Canada; 2 Bissett School of Business, Mount Royal University, Calgary, Alberta, Canada; New York University Abu Dhabi, UNITED ARAB EMIRATES

## Abstract

The naive approach to preventing discrimination in algorithmic decision-making is to exclude protected attributes from the model’s inputs. This approach, known as “equal treatment,” aims to treat all individuals equally regardless of their demographic characteristics. However, this practice can still result in unequal impacts across different groups. Recently, alternative notions of fairness have been proposed to reduce unequal impact. However, these alternative approaches may require sacrificing predictive accuracy. The present research investigates public attitudes toward these trade-offs in the United States. When are individuals more likely to support equal treatment algorithms (ETAs), characterized by higher predictive accuracy, and when do they prefer equal impact algorithms (EIAs) that reduce performance gaps between groups? A randomized conjoint experiment and a follow-up choice experiment revealed that support for the EIAs decreased sharply as their accuracy gap grew, although impact parity was prioritized more when ETAs produced large outcome discrepancies. Additionally, preferences polarized along partisan identities, with Democrats favoring impact parity over accuracy maximization while Republicans displayed the reverse preference. Gender and social justice orientations also significantly predicted EIA support. Overall, findings demonstrate multidimensional drivers of algorithmic fairness attitudes, underscoring divisions around equality versus equity principles. Achieving standards around fair AI requires addressing conflicting human values through good governance.

## Introduction

Algorithms increasingly guide important decisions about who has access to financing [1], how much people pay for insurance [2], and who should receive medical care [3,4]. A bank’s lending decision, for instance, might be based on a statistical risk assessment algorithm that predicts applicants’ likelihood of repaying the loan. As the use of algorithmic decision-making expands to other consequential domains, such as education, employment, and criminal justice, concerns have emerged that the underlying models may inadvertently perpetuate or even amplify human biases, resulting in discriminatory and inequitable outcomes [5–7]. For example, a widely used algorithm for assessing the risk of criminal re-offense (COMPAS) was found to produce biased predictions against African-American defendants [5], and Apple Card’s algorithm for predicting creditworthiness was found to produce worse outcomes for women than for men [1]. In response, many have called for more fairness in algorithmic decision-making [8–10].

The importance of this policy issue was evident when U.S. President Biden recently issued an executive order directing government agencies to combat algorithmic discrimination to ensure that AI advances equity and civil rights. The executive order adds to existing antidiscrimination laws that date back to the Civil Rights Act of 1964. These laws generally address two forms of discrimination: disparate treatment and disparate impact [11]. Disparate treatment occurs when individuals are intentionally treated differently based on protected class membership like race, gender, or disability. For algorithmic systems, this means directly using sensitive attributes to construct predictions or set thresholds is prohibited [11,12]. In contrast, disparate impact focuses on outcome discrimination and condemns practices that have uneven impacts on different groups [11].

The naive practice in algorithmic decision-making is to prevent disparate treatment by excluding protected attributes from inputs. This notion of fairness is known as equal treatment, as it seeks to treat individuals equally regardless of their demographic membership [13,14]. Nevertheless, excluding demographics from a model’s inputs can still lead to unequal impacts across groups if systematic differences exist along demographic lines [5,15,16]. For instance, a credit scoring model that doesn’t take race or gender as inputs but uses inputs like income level, debt levels, assets, zip code, etc., that are correlated with demographics may result in different approval rates for different groups.

Proposed alternatives aim to reduce disparate impact and promote greater parity of outcomes [17–19]. Unfortunately, attempts at equalizing impact may result in one or both of the following undesirable side effects. First, attempts to equalize impact may require treating different groups differently, which undermines the principle of equal treatment. Second, attempts at equalizing impact may unintentionally result in sacrificing predictive accuracy [12,20]. The difficulty of achieving impact parity without sacrificing performance and/or treatment parity has sparked policy debates among philosophers and legal scholars on whether and how to balance these goals in crafting fairness legislation for algorithmic systems [11,13,21–23].

Conspicuously missing from these debates, however, are the general public’s views on the matter. Yet knowledge of public opinion is critical for ensuring that any impactful policy decisions democratically incorporate the public’s voice. Political ideology likely plays a crucial role in shaping these preferences. Research shows that liberals and conservatives rely on different moral foundations when evaluating fairness and equity [24]. Left-leaning individuals tend to emphasize equality of outcomes and show greater concern for addressing historical disadvantages [25]. In contrast, right-leaning individuals typically prioritize individual merit and are more accepting of outcome disparities that result from standardized procedures [26]. These ideological differences extend to views on technological regulation and corporate oversight [27], suggesting that political affiliation may significantly influence how people evaluate accuracy-fairness trade-offs in algorithmic decision-making. In this research, we begin to bridge this gap by investigating people’s preferences for equal treatment algorithms (ETA) that tend to be more accurate versus equal impact algorithms (EIA) that are less accurate but aim to achieve impact parity.

## Why can’t we have it all?

Before delving into public preferences, we present a stylized example, adapted from [12], to illustrate why it is difficult to achieve equal impact and equal treatment simultaneously. Consider a bank evaluating loan applications. Suppose there are two groups of applicants (a regular group and a protected group). In each group, some applicants will pay back their loans (non-defaulters) and others will not (defaulters). The bank wishes to only grant loans to non-defaulters. However, it is not known in advance who will default and who will pay back their loan. So, the bank develops an algorithm to predict each applicant’s probability of paying back the loan and uses the same profit-maximizing threshold for both groups (say.55).

**Fig 1:**
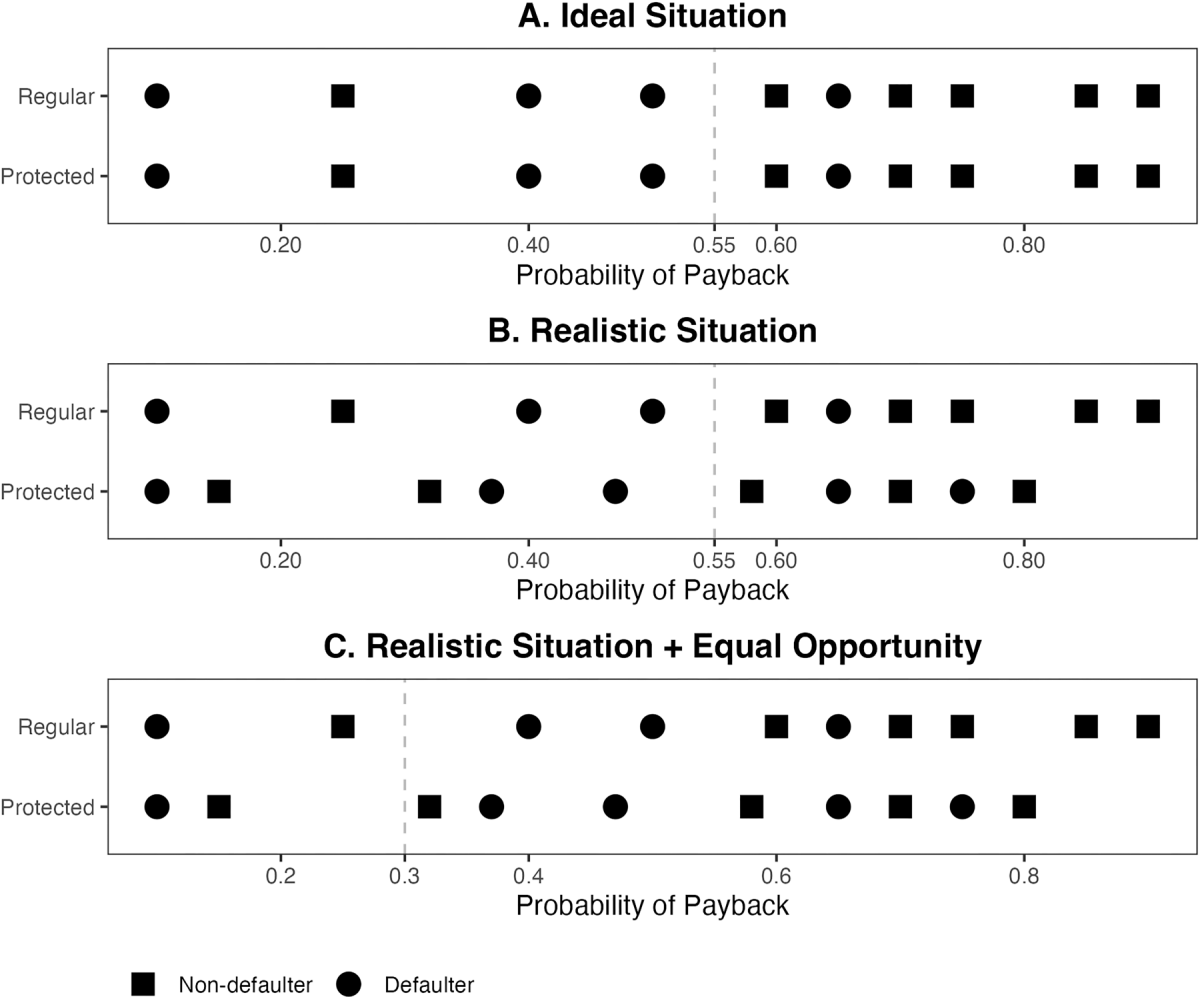
Algorithmic lending decision under ideal and realistic risk distributions.

In an ideal world, the risk of defaulting on a loan would be unrelated to group membership. That is, the estimated probability of payback would have the same distribution in the regular and in the protected groups (see Fig 1A). In this case, the algorithm, which is an ETA, can achieve both treatment parity (as it applies the same rule to everyone) and impact parity (as it leads to identical outcomes for both groups). However, such situations are rare. In reality, risk distributions vary between groups (see Fig 1B), often reflecting historical and social biases [11,18,20,28,29]. In such situations, the ETA fails to achieve impact parity. For one, it violates the notion of statistical parity [30], which requires that regular and protected groups have the same approval rate. In our example, the ETA approves 60% of the applicants in the regular group but only 50% of the applicants in the protected group. However, statistical parity is a rather crude measure of impact parity, as it fails to account for merit (i.e., the applicants’ creditworthiness). Unfortunately, the ETA also violates the more refined notion of equal opportunity [21], which requires that approval rates be similar among the qualified applicants (i.e., the non-defaulters) in both groups. In our example, the ETA approves 83% of the non-defaulters in the regular class but only 60% of the non-defaulters in the protected class.

Note that statistical parity and equal opportunity are two of many fairness principles that prioritize impact parity in algorithmic decision-making [19,31]. Past research [6,18] has shown that, under most circumstances, the various fairness principles cannot be achieved simultaneously. Nonetheless, equal opportunity is particularly compelling because it provides historically disadvantaged groups with equal access to opportunities [12,21]. We focus on equal opportunity as a representative notion of equal impact.

Equal opportunity can be achieved in our example by changing the threshold from .55 to .30 (see Fig 1C). Doing so would raise the non-defaulters’ approval rate in the protected class to 80%, bringing it in line with the 83% approval rate in the regular class. However, this gain in impact parity comes with a 15% loss in overall predictive accuracy. With the original threshold, the algorithm misclassifies two applicants in the regular group and four applicants in the protected group for a total of six errors and an overall accuracy rate of 70%. With the lower threshold, the algorithm misclassifies four applicants in the regular group and five applicants in the protected group for a total of nine errors and an overall accuracy rate of 55%.

The extent of accuracy loss can be mitigated by using different thresholds for the regular and protected groups. For example, applying the original threshold to the regular group and the lower threshold to the protected group would result in a total of seven classification errors and a 65% accuracy rate, which is only 5% lower than the most accurate model. However, doing so violates the doctrine of equal treatment and may result in legal challenges. More sophisticated fairness-enhancing techniques, such as constraint optimization and dynamic reweighting [32] can also be used, but they, too, require trading off some degree of predictive accuracy. In sum, predictive accuracy and equalized impact are simply different criteria, and optimizing for one inevitably leads to a suboptimal outcome for the other [20].

While the technical challenges of balancing accuracy and fairness are well documented, understanding public perceptions of these trade-offs has important implications for institutions deploying algorithmic decision systems. First, public opinion can significantly influence policy-making around algorithmic fairness, as evidenced by the EU AI Act’s development. Kieslich & Lunich [33] show how public concerns about discrimination and privacy have directly influenced regulatory approaches like algorithmic audits and bias testing requirements, noting that “the European Parliament and European Council as democratic representative institutions need to heed the demands of citizens to a certain extent (page 173).” This influence became particularly apparent in debates around regulating real-time biometric identification systems, where public concerns about privacy and discrimination substantially shaped policy provisions [33]. Public influence on AI policy is also evident in the United States, where advocacy organizations like the Center for Democracy & Technology and Consumer Reports played a crucial role in the passage of the Colorado Artificial Intelligence Act in 2024, which aims to protect individuals from algorithmic discrimination [34].

Second, institutions face reputational risks when their algorithms are perceived as unfair. As Draws et al. [35] demonstrate, public backlash against biased algorithms can damage brand value and erode customer trust, particularly in sensitive domains like financial services. The 2019 Apple Card controversy illustrates this dynamic, where public allegations of gender bias in credit limit algorithms triggered regulatory investigations and resulted in reputational damage [1]. Finally, many institutions now view algorithmic fairness as an essential component of corporate social responsibility rather than merely a technical or regulatory challenge. This shift reflects a growing recognition that ethical AI practices are crucial for maintaining public trust and long-term business sustainability [36]. Understanding public preferences regarding accuracy-fairness trade-offs can thus help institutions develop algorithms that not only perform well technically but also align with societal values and expectations.

## Equal treatment versus equal impact

Choosing between equal treatment algorithms characterized by higher predictive accuracy (ETA) and less accurate algorithms that prioritize impact parity (EIA) presents a moral dilemma between two desirable yet incompatible values. Moral dilemmas have been studied extensively in philosophy and moral psychology [37,38], including in the context of artificial intelligence [39,40]. These studies tend to focus on trolley-like problems, in which participants have the option of saving many people by sacrificing one person. For example, in the Moral Machine experiment [39], participants had to decide how they would want autonomous vehicles to solve trolley-like dilemmas in the context of unavoidable accidents. In this research, we focus on a different kind of dilemma – having to choose between prioritizing impact parity or predictive accuracy. Specifically, we investigate the following questions:

1. What algorithm attributes and features of the decision context most influence people’s preference for EIA vs. ETA?
2. Do the effects of the various attributes on algorithmic preference vary with individual differences in political affiliation and social justice orientations?
3. Do differences in demographic characteristics (gender, age, and ethnicity), political leanings, and social justice orientations directly influence preference for EIA vs. ETA?

To answer these questions, we conducted a paired-profile conjoint experiment [41] and a follow-up choice experiment. Conjoint experiments are particularly well-suited for analyzing trade-offs when choosing between options that simultaneously vary on multiple attributes [42]. We focused on seven scenario-level attributes: (1) the accuracy level of the ETA (three levels: 90%, 80%, 70%); (2) loss of accuracy if the EIA is adopted (three levels: 5%, 10%, 20%). In the survey, this attribute appears as the accuracy level of the EIA, which is automatically calculated based on the value of the first attribute; (3) the group disadvantaged by the ETA (three levels: Women, People of Colour, People with Disabilities); (4) the degree of disadvantage (three levels: 5%, 10%, 20%); (5) the severity of consequences (two levels: Moderate, Severe); (6) resource availability (two levels: Abundant, Scarce); and (7) algorithm currently in use (three levels: ETA, EIA, Neither).

Individual-level variations are also likely to affect algorithmic preference. For instance, people differ in their perceptions of distributive justice [43,44]. Fairness in algorithmic decision-making is closely tied to how valuable resources (e.g., financial loans and access to healthcare) are distributed among various groups. Three distinct principles of distributive justice have long been proposed: equality, equity, and need [45,46]. The principle of equality suggests that all individuals should have equal access to benefits and burdens. The principle of equity emphasizes the importance of distributing benefits and burdens proportionally and according to individual contributions and efforts. The principle of need invokes a selective concern for those who are most in need. Miller [47] added a fourth principle: entitlement, according to which benefits and burdens should be allocated based on ascriptive characteristics, such as one’s sex or social origin, or on status characteristics that have been attained previously, like occupational status. Entitlement differs from equity in that the allocation of benefits is not based on current efforts or contributions.

Individual differences in attitudes toward these four dimensions of distributive justice are captured by the Basic Social Justice Orientations (BSJO) scale [43]. We expect individuals who score high on the equality and need dimensions of the BSJO scale to show a higher preference for the EIA than those who score low. We also expect political affiliation to strongly influence algorithmic preference. As discussed earlier, political affiliation strongly influences views on equality versus efficiency trade-offs. Building on research showing systematic liberal-conservative differences in fairness perceptions [24], we expect Republicans, who tend to be more right-leaning, to be less concerned with equal impact and, hence, to display a lower preference for the EIA than Democrats, who are generally more left-leaning [27,48].

## Study 1: Conjoint experiment

### Methods

This research was approved by the University of Calgary Conjoint Faculties Research Ethics Board (REB18-1974) and was conducted according to the principles expressed in the Declaration of Helsinki. Written informed consent was obtained from all individual participants. The median completion time for Study 1 was 15 min and 46 seconds.

Using Prolific Academic’s representative sample feature, we recruited a sample of adult US residents between August 2 and August 5, 2023. The sample was quota-matched to the US general population in terms of age, gender, and ethnicity. We planned to recruit 2,000 participants but were unable to fill certain quotas (e.g., Asian females over 58 years of age). The final sample consisted of 1,975 participants (959 female, 981 male, 24 non-binary, 11 no answer; *M*_age_ =  44.8, *SD*_age_ =  15.0; 1,413 White, 250 Black, 112 Asian, 85 Hispanic, 115 Other). The data, analysis scripts, and study preregistration are available here.

**Fig 2:**
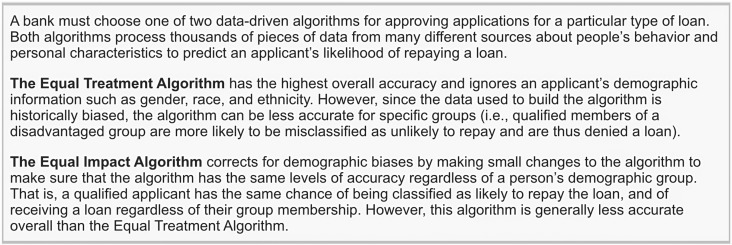
Sample scenario description.

We conducted a paired-profile conjoint experiment [41] to explore the factors that influence people’s preference for EIA vs. ETA. Each participant responded to three kinds of scenarios (Lending, Insurance, and Healthcare), with 4 random variations each. Thus, each participant made decisions on 12 different profiles, resulting in a total of 23,700 random cases. Each case showed a brief description of a decision-making scenario (an example can be found in Fig 2), followed by a table with the randomly generated seven attributes (an example can be found in Table 1). The three scenario types and the descriptions of the ETA and EIA were presented in random order at the respondent level.

**Table 1:** Sample attribute table.

Attribute	Attribute level
1) Equal Treatment Algorithm Accuracy	The Equal Treatment Algorithm has an Overall Accuracy of **90%**.
2) Equal Impact Algorithm Accuracy	The Equal Impact Algorithm has an Overall Accuracy of **80%**.
3) Group Disadvantaged by the Equal Treatment Algorithm	The Equal Treatment Algorithm disadvantages **women**.
4) Degree of Disadvantage	Under the Equal Treatment Algorithm, women are **20%** more likely to be misclassified than men.
5) Severity of Consequences	If denied this type of loan, customers usually face **severe financial difficulties (e.g., foreclosure, eviction, poverty)**
6) Availability	The supply of this type of loan is **abundant.** Because they are very safe, and easy to administer, the loan can be offered to many.
7) Current Algorithm	The bank is **currently using the Equal Impact Algorithm** and is trying to decide whether to keep using it in the future or to switch to the Equal Treatment Algorithm.

**Fig 3:**
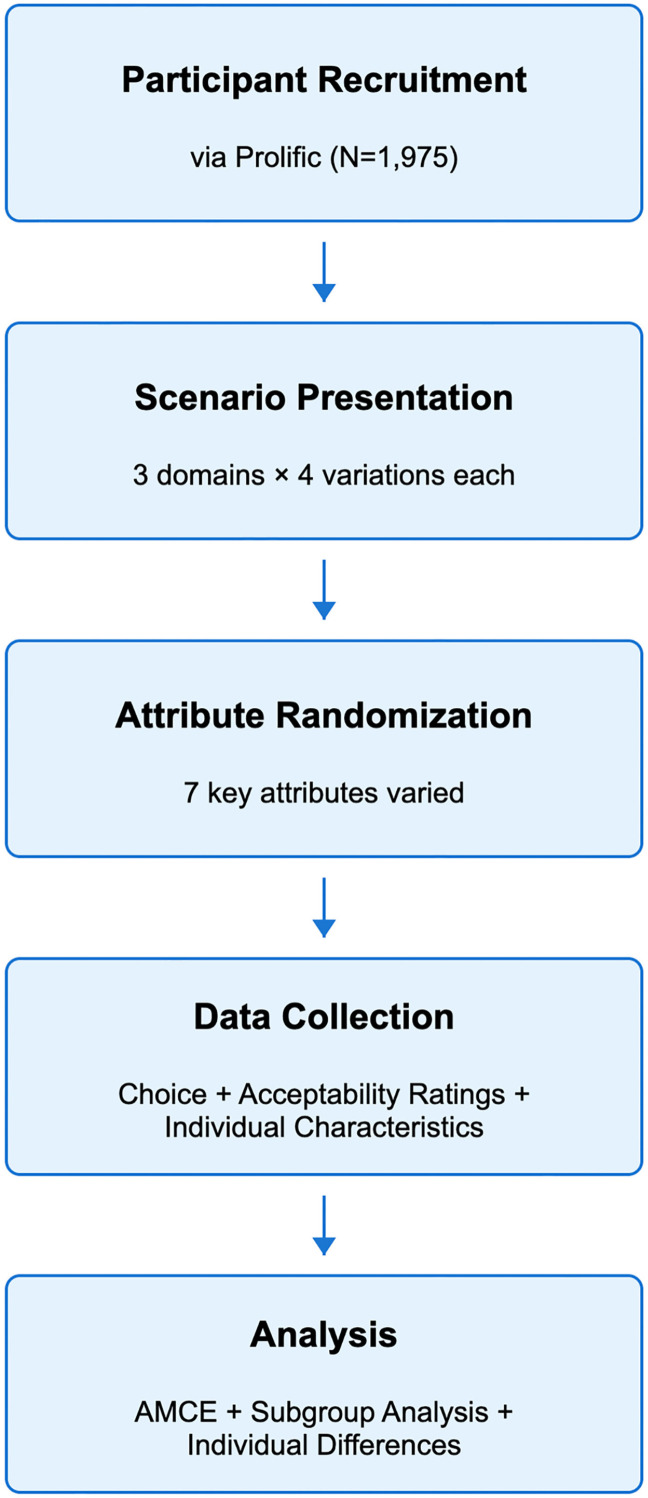
Methodological flowchart.

We performed power analyses using the R package cjpowR [49] to determine the power of our design given our sample size and two minimum effect sizes of interest: Average Marginal Component Effect (AMCE) =  0.025 across scenarios, and AMCE =  0.050 within each scenario. For n =  23,700 (1,975 *  12), alpha = .05, and a maximum number of attribute levels =  3, our design has 88% power to detect a small effect size AMCE =  0.025 across scenarios. For n =  7,900 (1,975 *  4), alpha = .05, and a maximum number of attribute levels =  3, our design has 95% power to detect a medium effect size AMCE =  0.050 within each scenario. A visual representation of the study design is shown in Fig 3.

### Measures

The main outcome was a binary choice (EIA vs. ETA) in response to the question: “Which algorithm should the company use going forward?” Participants also rated the acceptability of each algorithm on a 5-point scale (1 =  Totally Unacceptable, 5 =  Perfectly Acceptable). After responding to one profile, they proceeded to the next profile until they completed all 12 rounds.

Following the conjoint task, participants completed the Basic Social Justice Orientation (BSJO) scale [43], consisting of a 12-item, 5-point Likert scale (1 =  Strongly Disagree, 5 =  Strongly Agree) assessing participants’ opinions on how a society can be fair and just (see supplementary materials for complete scale). The equality, need, equity, and entitlement dimensions were measured by three items each (Cronbach’s alpha: equality = .78; need = .70; equity = .72; entitlement = .77). To evaluate the structural validity of the BSJO dimensions, we conducted a confirmatory factor analysis comparing our hypothesized four-factor structure against a single-factor model. The four-factor model demonstrated significantly better fit (Δχ2(6) =  34,876.32, p < .001) with superior indices (CFI = .92, TLI = .89, RMSEA = .09, SRMR = .06) compared to the single-factor solution (CFI = .57, TLI = .47, RMSEA = .18, SRMR = .15). The substantial improvement in fit provides strong evidence for the BSJO dimensions as empirically distinct constructs.

After the BSJO, participants identified their federal political party affiliation (Democrat, Republican, Independent, Other). For analysis purposes, we combined the Independent and Other categories due to the small number of respondents who selected Other. Lastly, they indicated their gender (Male, Female, Non-Binary, Prefer not to answer), age, ethnicity (select all: American Indian or Alaska Native, Asian, Black or African American, Hispanic or Latino or Spanish Origin, Native Hawaiian or Other Pacific Islander, White, Other), and disability status (Yes, No, Prefer not to answer). For analysis purposes, on the gender measure, we combined the Non-Binary and Prefer not to answer into a single Other category. We also recoded the ethnicity variable into 5 categories: Asian, Black, Hispanic, White, and Other (including people who selected Native Hawaiian or Other Pacific Islander, Other, or more than one ethnicity).

## Results

### Descriptive analysis

**Fig 4:**
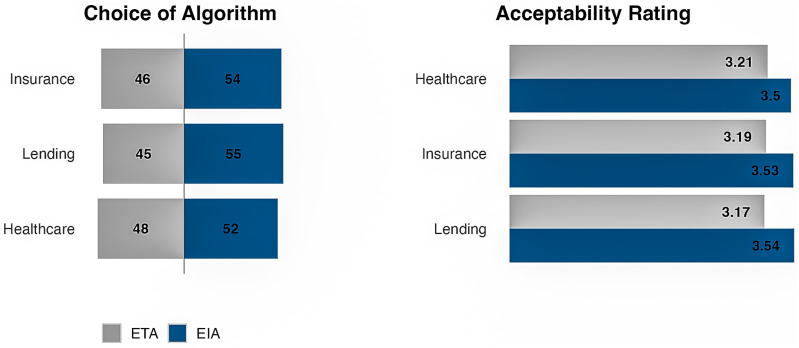
Preference for the EIA vs. ETA across scenarios.

Overall, respondents in our sample selected the EIA in 53.5% of the 23,700 cases. In the Lending scenario, the choice share of the EIA was 54.8%. In the Insurance scenario, it was 53.9%, and in the Healthcare scenario, it was 51.9% (Fig 4). Both algorithms were generally rated as sufficiently acceptable (above the mid-point of the acceptability scale), though, on average, the EIA was rated as more acceptable than the ETA in all three scenarios (Fig 4). Because the patterns of results for acceptability mirror those for choice, the remainder of the paper focuses on choice as the outcome measure.

The choice results are rounded to the nearest percent. The acceptability ratings are on a 5-point scale (1 =  Totally Unacceptable, 5 =  Perfectly Acceptable).

### Conjoint analysis: What factors influence algorithmic choice?

We estimated the average marginal component effect (AMCE) of each attribute in our conjoint experiment [50] using the R package cregg [51]. The AMCE represents the effect of a particular attribute averaged over the joint distribution of the remaining attributes [41,52]. We also estimated marginal means to facilitate the interpretation of the attributes’ effects.

**Fig 5:**
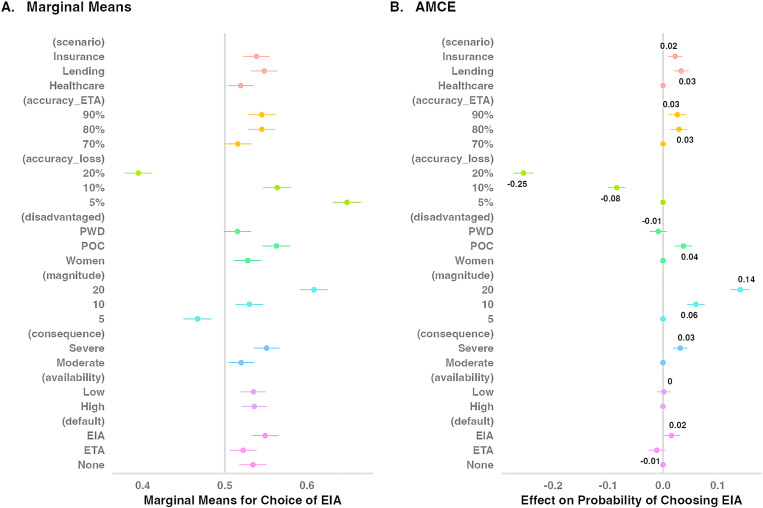
Preference for the EIA – conjoint results.

Fig 5 shows the marginal means and the AMCEs for the choice outcome, pooled across all three scenarios. The largest effects on the probability of choosing the EIA came from accuracy loss and magnitude of disadvantage (see also Tables 2 and 3). The probability of choosing the EIA was estimated at .65 [.63,.67] when it led to a relatively small loss in accuracy (i.e., when it was 5% less accurate than the ETA). In contrast, when the EIA was 10% less accurate than the ETA, the probability of choosing it decreased to .56 [.55,.58] (AMCE =  -0.08, 95% CI =  [-0.10, -0.07], *z* =  -10.32, *p* < .001). When the EIA was 20% less accurate than the ETA, the probability of choosing it dropped to .39 [.37,.41] (AMCE =  -0.25 [-0.27, -0.24], *z* =  -27.06, *p* < .001). Thus, it appears that people were willing to sacrifice a modest amount of accuracy to ensure equal impact. However, larger losses in accuracy were deemed less acceptable.

**Table 2:** Marginal means – Pooled across scenarios.

Attribute	Level	MM	SE	Lower CI	Upper CI
Scenario	Healthcare	0.52	0.01	0.50	0.54
Scenario	Lending	0.55	0.01	0.53	0.56
Scenario	Insurance	0.54	0.01	0.52	0.55
ETA Accuracy	70%	0.52	0.01	0.50	0.53
ETA Accuracy	80%	0.55	0.01	0.53	0.56
ETA Accuracy	90%	0.54	0.01	0.53	0.56
Accuracy Loss	5%	0.65	0.01	0.63	0.67
Accuracy Loss	10%	0.56	0.01	0.55	0.58
Accuracy Loss	20%	0.39	0.01	0.37	0.41
Disadvantaged Group	Women	0.53	0.01	0.51	0.54
Disadvantaged Group	POC	0.56	0.01	0.55	0.58
Disadvantaged Group	PWD	0.52	0.01	0.50	0.53
Magnitude	5%	0.47	0.01	0.45	0.48
Magnitude	10%	0.53	0.01	0.51	0.55
Magnitude	20%	0.61	0.01	0.60	0.63
Consequence	Moderate	0.52	0.01	0.50	0.54
Consequence	Severe	0.55	0.01	0.54	0.57
Availability	High	0.54	0.01	0.52	0.55
Availability	Low	0.53	0.01	0.52	0.55
Default	None	0.53	0.01	0.52	0.55
Default	ETA	0.52	0.01	0.51	0.54
Default	EIA	0.55	0.01	0.53	0.57

**Table 3:** AMCEs – Pooled across scenarios.

Attribute	Level	AMCE	SE	*z*	*p*	Lower CI	Upper CI
Scenario	Healthcare	0.00					
Scenario	Lending	0.03	0.01	4.81	<.001	0.02	0.05
Scenario	Insurance	0.02	0.01	3.29	<.001	0.01	0.04
ETA Accuracy	70%	0.00					
ETA Accuracy	80%	0.03	0.01	3.73	<.001	0.01	0.04
ETA Accuracy	90%	0.03	0.01	3.22	.001	0.01	0.04
Accuracy Loss	5%	0.00					
Accuracy Loss	10%	−0.08	0.01	−10.32	<.001	−0.10	−0.07
Accuracy Loss	20%	−0.25	0.01	−27.05	<.001	−0.27	−0.24
Disadvantaged Group	Women	0.00					
Disadvantaged Group	POC	0.04	0.01	4.69	<.001	0.02	0.05
Disadvantaged Group	PWD	−0.01	0.01	−1.06	.29	−0.02	0.01
Magnitude	5%	0.00					
Magnitude	10%	0.06	0.01	7.39	<.001	0.04	0.08
Magnitude	20%	0.14	0.01	15.83	<.001	0.12	0.16
Consequence	Moderate	0.00					
Consequence	Severe	0.03	0.01	4.70	<.001	0.02	0.04
Availability	High	0.00					
Availability	Low	0.00	0.01	0.24	.81	−0.01	0.01
Default	None	0.00					
Default	ETA	−0.01	0.01	−1.50	.13	−0.03	0.00
Default	EIA	0.02	0.01	1.94	.05	0.00	0.03

The degree of disadvantage under equal treatment also had a large impact on participants’ algorithmic preference. When the ETA produced a relatively small disadvantage (i.e., when applicants from a disadvantaged group were 5% more likely to be misclassified than those from a regular group), the probability of choosing the EIA was .47 [.45,.48]. However, when the ETA produced a larger disadvantage (i.e., a 10% discrepancy in misclassification), the probability of choosing the EIA increased to .53 [.51,.55] (AMCE =  0.06 [0.04, 0.08], *z* =  7.39, *p* < .001). When the discrepancy in misclassification was even larger (i.e., 20%), the probability of selecting the EIA increased to .61 [.60,.63] (AMCE =  0.14 [0.12, 0.16], *z* =  15.83, *p* < .001).

Several other conjoint features had modest but statistically significant effects on the likelihood of choosing the EIA. For example, compared to healthcare as the reference scenario, the lending scenario led to a 3% increase in the probability of choosing the EIA (AMCE =  0.03 [0.02, 0.05], *z* =  4.81, *p* < .001), while the insurance scenario led to a 2% increase in the probability of selecting the EIA (AMCE =  0.02 [0.01, 0.04], *z* =  3.29, *p* < .001).

Changing the consequence attribute from moderate to severe increased the probability of choosing the EIA by 3 percentage points (AMCE =  0.03 [0.02, 0.04], *z* =  4.70, *p* < .001). Likewise, changing the disadvantaged group from women to people of color and ethnic minorities increased the probability of choosing the EIA by 4 percentage points (AMCE =  0.04 [0.02, 0.05], *z* =  4.69, *p* < .001).

Changing the disadvantaged group from women to people with disabilities resulted in a statistically non-significant decrease in the probability of choosing the EIA (AMCE =  -0.01 [-0.02, 0.01], *z* =  -1.06, *p* =  0.29). Similarly, changing the resource availability from abundant to scarce had no significant impact on the probability of choosing the EIA (AMCE =  0.002 [-0.01, 0.01], *z* =  0.24, *p* =  0.81). Finally, changing the baseline accuracy of the ETA also influenced algorithmic preference. Compared to a 70% ETA accuracy rate, an 80% ETA accuracy rate led to a 3% increase in the probability of choosing the EIA (AMCE =  0.03 [0.01, 0.04], *z* =  3.73, *p* < .001). A 90% ETA accuracy rate also led to a 3% increase in the probability of selecting the EIA (AMCE =  0.03 [0.01, 0.04], *z* =  3.22, *p* = .001).

### Conjoint analyses by scenario

**Fig 6:**
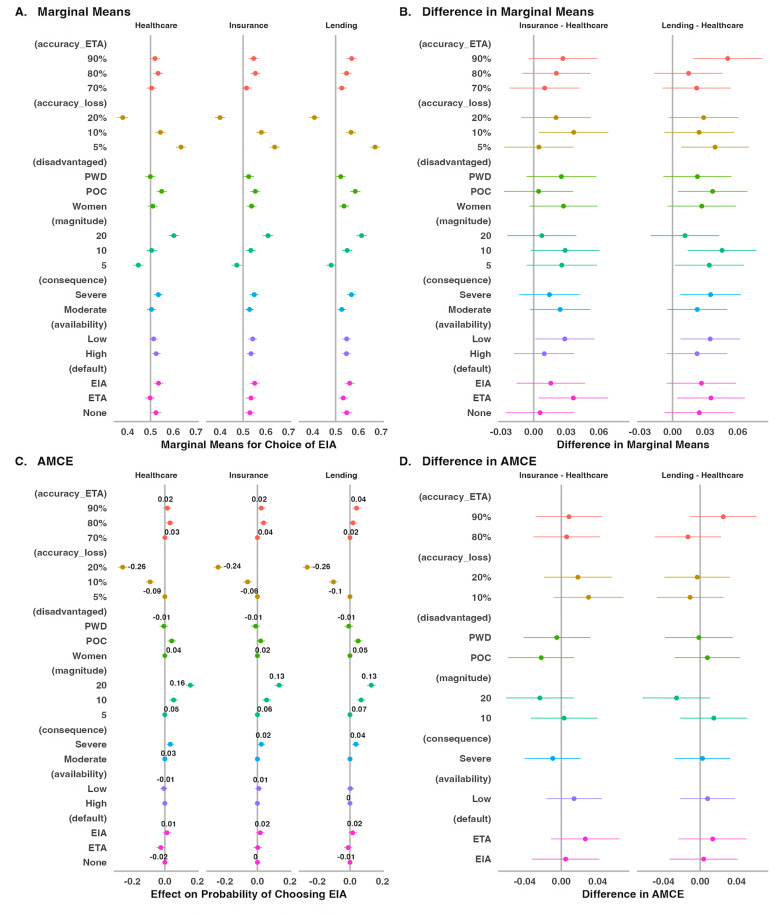
Preference for the EIA – conjoint results by scenario.

The preceding analysis revealed that the probability of choosing the EIA was slightly higher in the lending and insurance scenarios than in the healthcare scenario. A related question is whether the effects of the various conjoint features may differ between scenarios. To answer this question, we compared the conditional effects of the attributes in each scenario. Fig 6 shows that while the conditional marginal means at some attribute levels were higher in the lending scenarios (e.g., 90% ETA accuracy and 5% accuracy loss) and insurance (e.g., 10% accuracy loss and ETA as the default algorithm) than in the healthcare scenario (panels A and B), there were no significant differences in any of the attributes’ effects (conditional AMCEs) on the probability of choosing the EIA (panels C and D). Consistent with the pooled data, the largest effects on the probability of choosing the EIA in each scenario, came from accuracy loss and magnitude of disadvantage (see also Tables S2A–S5B in the supplementary materials in S1 File).

### Conjoint analyses by political affiliation and social justice orientation

We conducted subgroup analyses to examine how the various conjoint attributes might affect algorithmic preference differently, depending on respondents’ political affiliations and social justice orientations.

**Fig 7:**
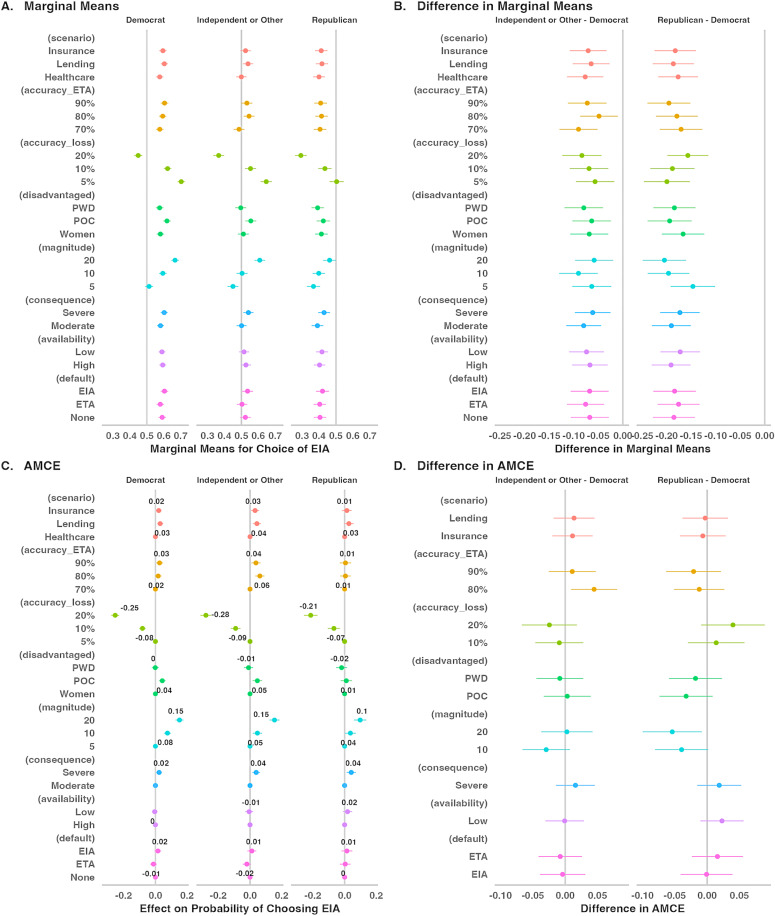
Subgroup analysis – Political affiliation.

Fig 7A shows the conditional marginal means for the three political affiliation groups. Democrats were more likely to choose the EIA over the ETA at almost all attribute levels. The only attribute level that made Democrats prefer the ETA over the EIA was when the EIA led to a 20% loss of accuracy. In contrast, Republicans preferred the ETA over the EIA at all attribute levels, except when the EIA led to only a 5% loss of accuracy. At that level, Republicans were indifferent between the two algorithms. Independents showed more sensitivity to the different attributes in their algorithmic preferences. They preferred the EIA at certain attribute levels (e.g., 5% and 10% loss of accuracy, and 20% magnitude of disadvantage), preferred the ETA at other attribute levels (e.g., 20% accuracy loss and 5% magnitude of disadvantage), and were indifferent at other attribute levels (e.g., 10% magnitude of disadvantage and moderate consequences).

Fig 7B shows that Democrats were significantly more likely to choose the EIA than both Independents and Republicans at all attribute levels. Fig 7C shows that accuracy loss and magnitude of disadvantage had the largest effects on the probability of choosing the EIA for all three groups. Fig 7D shows the effects of most attributes did not differ significantly between Democrats and Independents and only the effect of the magnitude of disadvantage differed between Democrats and Republicans. Changing the magnitude of disadvantage from 5% to 10% and from 5% to 20% had a smaller effect on Republicans than on Democrats’ choices (see also Tables S6A–S9B in supplementary materials S1 File).

We also conducted subgroup analyses on each dimension of the BJSO scale (see Figures S1–S4, and Tables S10–S25 in the supplementary materials S1 File). Across all four dimensions, accuracy loss and magnitude of disadvantage had the largest effects on the probability of choosing the EIA. Participants who scored high on equality and need orientations were more likely to prefer the EIA over the ETA at all attribute levels, except when the accuracy loss was 20% or when the magnitude of disadvantage was 5%. Participants who scored high on equity orientation only preferred the EIA when accuracy loss was 10% or 5% and when the magnitude of disadvantage was 20%. These participants were largely indifferent between the two algorithms at most attribute levels but favored the ETA when accuracy loss was 20% and when the magnitude of disadvantage was 5%. Participants who scored high on entitlement orientation preferred the EIA only when the accuracy loss was 5%. They preferred the ETA at most attribute levels and were indifferent between the algorithms at a few attribute levels (e.g., when accuracy loss was 10% or when the magnitude of disadvantage was 20%).

### Demographic and individual differences in algorithmic preference

Subgroup analyses show that political affiliations and social justice orientations did not significantly influence the effect of most attributes on the probability of choosing the EIA. However, these analyses also suggested differences in algorithmic preferences between these groups at most attribute levels. In this section, we report on formal tests of the effects of individual characteristics, including gender, age, ethnicity, political affiliation, and social justice orientations, on algorithmic preferences averaged across the levels of attributes and scenarios.

Given the structure in our data (each participant provided 4 choices in each of 3 scenarios for a total of 12 responses), we fitted a series of generalized mixed models (one for each individual characteristic) with algorithmic choice as the binary outcome variable (0 =  ETA, 1 =  EIA); random intercepts for participants and scenarios; and fixed effects for the individual characteristics. We also included the seven conjoint attributes as covariates in the models. The models were estimated using the Laplace Approximation to maximum likelihood and implemented using the lme4 R package [53].

**Fig 8:**
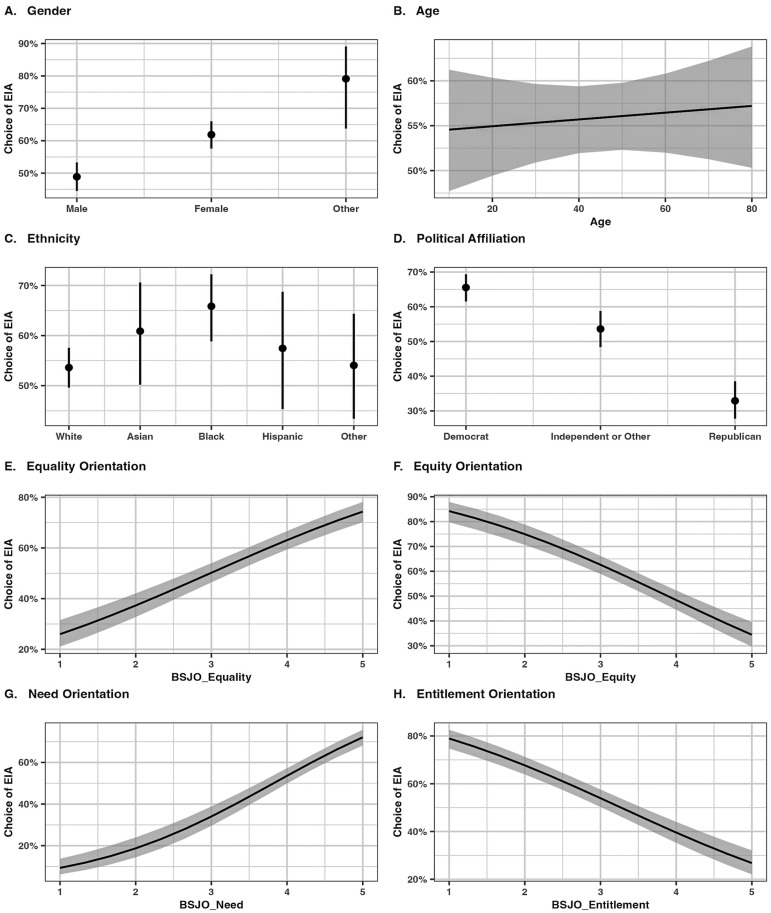
Probability of choosing the EIA by individual characteristics.

Gender had a significant influence on algorithmic choice. The probability of choosing the EIA was higher for females (Prob = .62 [.58,.66]) than for males (Prob = .49 [.44,.53], *z* =  5.18, *p* < .001, OR =  1.70). It was also higher for those who identified as Other (Prob = .79 [.64,.98], *z* =  3.49, *p* = .001, OR =  3.96) than for males (Fig 8A). Age did not significantly influence algorithmic choice (*b* = .002, *SE* = .003, *z* = .453, *p* = .65, OR =  1.00; Fig 8B). As for ethnicity, the only significant difference was between participants who identified as Black and those who identified as White (Fig 8C). On average, Black participants were more likely to choose the EIA (Prob = .66 [.59,.72]) than White participants (Prob = .54 [.50,.58], *z* =  3.32, *p* = .004, OR =  1.67).

Consistent with the patterns observed in the subgroup analysis, political affiliation had a large effect on algorithmic choice. As seen in Fig 8D, Republicans were much less likely to choose the EIA (Prob = .33 [.28,.38]) than Democrats (Prob = .66 [.62,.69], *z* =  -10.23, *p* < .001, OR = .26). Independents were also less likely to choose the EIA than Democrats (Prob = .54 [.48,.59], *z* =  -4.31, *p* < .001, OR = .61). Also consistent with the subgroup analyses, algorithmic preference was impacted by participants’ social justice orientations. Equality orientation (*b* = .53, *SE* = .05, *z* =  10.93, *p* < .001, OR =  1.70) and need orientation (*b* = .81, *SE* = .07, *z* =  12.04, *p* < .001, OR =  2.24) positively affected the probability of choosing the EIA (Fig 8E and 8G). In contrast, equity orientation (*b* =  -.58, *SE* = .06, *z* =  -10.52, *p* < .001, OR = .56) and entitlement orientation (*b* =  -.58, *SE* = .05, *z* =  -11.56, *p* < .001, OR = .56) negatively affected the probability of choosing the EIA (Fig 8F and 8H).

## Discussion

A key finding from the conjoint study is that participants balanced algorithmic accuracy against impact parity. Across domains, increases in accuracy loss led to lower preferences for the EIA, while increases in impact disparity produced stronger preferences for the EIA. This suggests a willingness to trade off some algorithmic accuracy to achieve greater equity in outcomes. However, it remains unclear whether participants in the conjoint study truly understood the quantitative implications of these trade-offs. Indeed, the abstract description of some attributes (e.g., women are 20% more likely to be misclassified than men) and the use of percentages rather than absolute numbers may have made it difficult for participants to fully appreciate the real-world implications of these differences. To address these limitations, we conducted a follow-up experiment using more concrete scenarios with absolute numbers (instead of percentages), clear specification of error direction, and explicit information about the consequences for both institutions and individuals.

## Study 2: Choice experiment

### Methods

We aimed to recruit a sample of 600 adult US residents on Prolific. Six hundred and sixty-six participants clicked on the study link, and 594 successfully passed the required comprehension checks and completed the study (343 female, 241 male, 7 non-binary, 3 no answer; *M*_age_ =  37.7, *SD*_age_ =  11.9; 356 White, 77 Black, 61 Asian, 39 Hispanic, 61 Other).

The study had a 5 (accuracy loss: 2%, 5%, 10%, 15%, 20%) ×  3 (impact disparity: 4%, 10%, 20%) mixed design, with accuracy loss manipulated within subjects and impact disparity between subjects. Participants first read detailed information about two types of loan approval algorithms. The Equal Treatment Algorithm (ETA) was described as excluding protected attributes from its inputs but potentially producing unequal impacts across groups due to historical biases in the data. The Equal Impact Algorithm (EIA) was described as making adjustments to ensure equal impact across groups but at the cost of lower overall accuracy (see the supplementary materials for complete descriptions).

Before proceeding to the choice tasks, participants had to pass three comprehension check questions: (1) “The Equal Impact Algorithm is typically more accurate than the Equal Treatment Algorithm” [False], (2) “The Equal Treatment Algorithm impacts women more negatively than men” [True], and (3) “Higher accuracy leads to higher profits for the bank” [True]. Participants who failed any one question had one additional attempt. Those failing both attempts were excluded from the study.

Each participant then made five choices between pairs of algorithms in a random order. The ETA was held constant within each impact disparity condition, correctly classifying 90 out of 100 applicants but with different error rates for men and women. For example, in the 20% disparity condition: “Out of each 100 qualified men, 5 are wrongly denied loans (5% wrong denials). Out of each 100 qualified women, 25 are wrongly denied loans (25% wrong denials).” The EIA achieved equal error rates across groups (15% for both men and women) but with varying levels of overall accuracy (88%, 85%, 80%, 75%, or 70%), corresponding to accuracy losses of 2%, 5%, 10%, 15%, and 20%).

The scenarios explicitly noted institutional implications (“For the bank, loan approval accuracy is closely tied to its financial performance. Higher accuracy leads to higher loan repayment rates and profitability. At the same time, achieving impact parity across demographic groups can affect the bank’s reputation and long-term customer relationships”) and individual consequences (“Being wrongly denied a loan can have significant negative consequences for individuals, potentially affecting their ability to buy homes, start businesses, or invest in education.”). After the algorithm choices, participants completed the Basic Social Justice Orientations scale and demographic questions, including political affiliation, as in the main study. The median completion time for Study 2 was 6 min and 59 seconds.

## Results and discussion

We excluded the 20% accuracy loss condition from the analysis due to a typographical error in the survey where the ETA wrong denial rate was incorrectly displayed as 917% instead of 17% in the 4% impact disparity scenario. Results of the analysis of the full dataset are reported in Table S26 and Figure S6 in the supplementary materials in S1 File.

To examine how progressive increases in accuracy loss and impact disparity influence algorithmic preference, we contrast-coded both variables using repeated contrasts. To determine the optimal model structure, we compared two mixed-effects logistic regression models: a simpler model with main effects only (accuracy loss and impact disparity as fixed effects, with random intercepts for participants) and a more complex model that included interaction terms between accuracy loss and impact disparity. While the likelihood ratio test indicated that the model including interactions provided a significantly better fit (χ²(6) =  21.99, *p* = .001), several considerations led us to select the more parsimonious main effects model.

First, although the complex model showed a lower AIC (2527.7 vs. 2537.6), the BIC, which more strongly penalizes model complexity, favored the simpler model (2578.1 vs. 2602.7). This suggests that the improvement in model fit may not justify the additional complexity introduced by the interaction terms. Second, an examination of the interaction coefficients revealed that most (5/6) were not statistically significant (all *p*s > .25), with only one term showing significance (*p* = .004). In contrast, the main effects remained robust and significant across both models, with similar magnitude and direction of effects. Given these considerations, we proceeded with the more parsimonious model for our final analyses.

**Fig 9:**
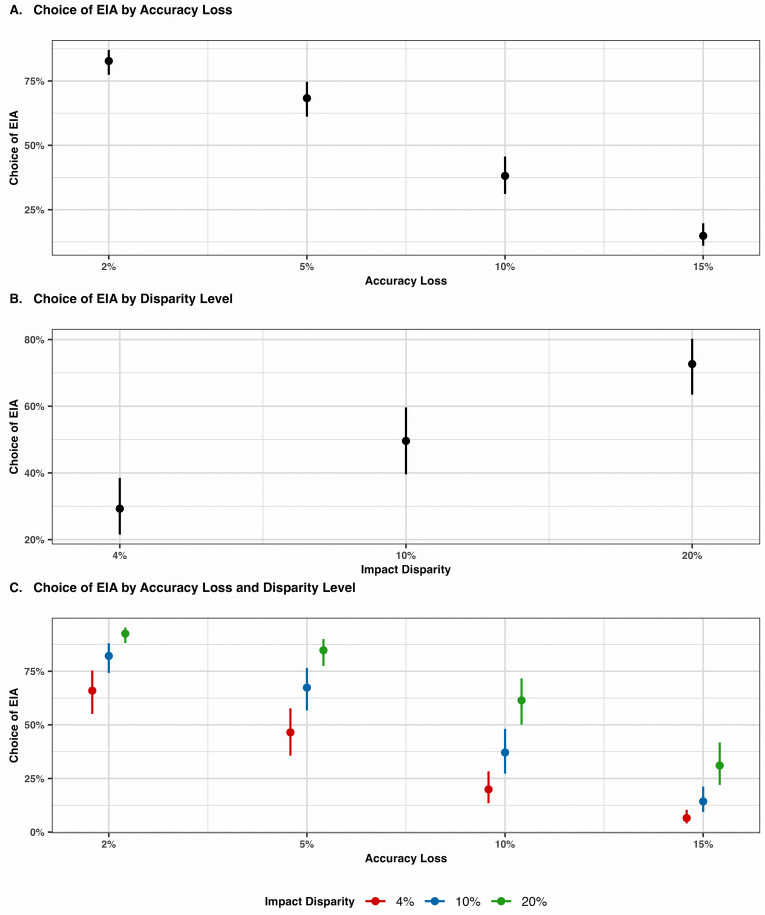
Effects of accuracy loss and impact disparity on choice of the EIA.

The analysis revealed a significant effect of accuracy loss on algorithmic choice, with a decreasing likelihood of selecting the EIA as the magnitude of accuracy loss increased (Fig 9 and Table 4). The predicted probability of choosing the EIA was highest at 2% accuracy loss (.83, [.77,.87]), decreased to .68 [.61,.75] at 5% loss, further declined to .38 [.31,.46] at 10% loss, and was lowest at 15% loss (.15 [.11,.20]). Comparing consecutive levels, 5% vs. 2% accuracy loss showed an odds ratio (OR) of .45 [.32,.63], *p* < .001), 10% vs. 5% showed OR = .29 [.20,.40], *p* < .001), and 15% vs. 10% showed OR = .28 [.20,.40], p < .001).

**Table 4:** Fixed effects for the choice of EIA from the mixed-effects logistic regression model.

Term	b (SE)	OR [95% CI]	p-value
(Intercept)	0.03 (0.12)	1.03 [0.81, 1.30]	.828
Accuracy Loss [5% vs. 2%]	−0.80 (0.17)	0.45 [0.32, 0.63]	<.001
Accuracy Loss [10% vs. 5%]	−1.25 (0.17)	0.29 [0.20, 0.40]	<.001
Accuracy Loss [15% vs. 10%]	−1.26 (0.18)	0.28 [0.20, 0.40]	<.001
Impact Disparity [10% vs. 4%]	0.87 (0.30)	2.38 [1.33, 4.24]	0.003
Impact Disparity [20% vs. 10%]	0.99 (0.30)	2.70 [1.50, 4.86]	<.001

In contrast, increased levels of impact disparity led to higher preferences for the EIA. The predicted probability of choosing the EIA was .29 [.22,.39]) in the 4% disparity condition, increased to .50 [0.40, 0.60] in the 10% condition (OR =  2.38, [1.33, 4.25], *p* = .003), and was highest at.73 [.64,.80]) in the 20% condition (OR =  2.70, [1.50, 4.86], *p* < .001). The random effects structure indicated substantial participant-level variance (*σ²* =  5.97), suggesting considerable individual differences in choice patterns.

Consistent with the main study’s findings, males (Prob = .36 [.28,.45]) were significantly less likely to choose the EIA than females (Prob = .60 [.52,.67], *z* =  -3.87, *p* < .001, OR = .38), while Democrats were much more likely to choose the EIA (Prob = .66 [.58,.74]) than both Republicans (Prob = .34 [.23,.46], *z* =  4.14, *p* < .001, OR =  3.89) and Independents (Prob = .41 [.32,.51], *z* =  3.85, *p* < .001, OR = 2.84). Moreover, preference for the EIA was positively associated with equality orientation (*b* = .65, *SE* = .13, *z* =  5.30, *p* < .001, OR =  1.92) and need orientation (*b* = .83, *SE* = .17, *z* =  4.95, *p* < .001, OR =  2.30) and negatively associated with equity orientation (*b* =  -.92, *SE* = .15, *z* =  -6.22, *p* < .001, OR = .40) and entitlement orientation (*b* =  -1.11, *SE* = .14, *z* =  -7.89, *p* < .001, OR = .33).

## General discussion

The use of data-driven algorithms for consequential decisions like lending, insurance, and medicine has raised concerns about potential biases. Typically designed to maximize accuracy, these algorithms often perpetuate unequal outcomes across demographic groups due to biases in the underlying data. Some have argued that algorithms should instead prioritize parity in impact across groups, even if this may decrease overall predictive performance. Indeed, achieving both impact parity and high accuracy has proven difficult, sparking policy debates among scholars. However, public perspectives are notably lacking in these discussions of algorithmic fairness.

This research makes three important contributions. First, it provides direct evidence of public views to inform debates on designing fair algorithms. These insights suggest that accuracy and impact parity should be balanced. Second, it reveals divisions in preferences that policymakers must grapple with through further political analysis. Third, it highlights the value-laden nature of determining appropriate algorithmic behavior and the need for values-sensitive design [54]. Overall, a shared understanding of what constitutes ethical AI is needed before setting technical standards.

Our investigation of public perceptions on the trade-offs between equal treatment algorithms that maximize predictive accuracy (ETA) versus equal impact algorithms that aim to achieve parity across groups (EIA) yielded three key insights. First, we found that most participants were willing to accept some loss in overall accuracy to ensure fairer outcomes. However, preference for the EIA decreased sharply as the accuracy gap widened, suggesting that accuracy is highly valued. Additionally, the preference for equalizing impact increased with the perceived degree of bias under the ETA. The effects of accuracy loss and bias magnitude were most influential across decision scenarios and subgroups.

Second, the study revealed ideological divides in attitudes toward algorithmic fairness. Liberals favored impact parity over accuracy maximization except when the accuracy loss was very large. In contrast, conservatives prioritized accuracy except when disparate impact was extremely high. This aligns with moral foundations theory [24] and reflects broader partisan disagreements over egalitarian values. It suggests that achieving societal consensus on regulating algorithmic fairness may be challenging. Still, the facts that conservatives did prefer the EIA given sufficient inequality, and democrats preferred ETA given sufficient accuracy, highlights potential common ground. An opportunity may exist to bring opposing sides together by emphasizing the societal benefits of mitigating extreme unfairness.

Finally, social justice orientations also predicted preferences. Those embracing equality and need principles strongly preferred the EIA, while those endorsing equity and entitlement principles favored accuracy. Interestingly, equity-oriented individuals supported the EIA more than entitlement-oriented individuals, suggesting that not all conservative principles oppose impact parity equally. Other individual differences like gender and ethnicity also impacted preferences. The finding that participants from historically disadvantaged groups (e.g., those identifying as female or Black) preferred the EIA more aligns with self-interest theories around fairness attitudes [55,56].

Our findings have important implications for both financial institutions and their customers. From an institutional perspective, the results provide concrete guidance for algorithm deployment decisions. While the public generally accepts some accuracy-fairness trade-offs, support for accuracy-maximizing algorithms drops sharply when disparate impacts become substantial. Specifically, when the difference in misclassification rates between groups reaches 20%, small sacrifices in accuracy (2-5%) to achieve impact parity receive strong public support. This suggests institutions should carefully monitor disparate impacts and consider adjustments when group differences exceed certain thresholds, even at the cost of modest accuracy reductions.

Our findings have equally important implications beyond financial services, particularly in healthcare and employment contexts where algorithmic decisions significantly impact human welfare. In healthcare settings, the stakes of accuracy-fairness trade-offs are especially high, as algorithmic decisions can directly affect mortality and morbidity outcomes. For instance, a widely used algorithm for predicting which patients need extra care was found to systematically underestimate the health needs of Black patients, potentially exacerbating existing health disparities [57]. Our finding that public support for accuracy-maximizing algorithms drops sharply when disparate impacts exceed certain thresholds suggests healthcare providers may face pressure to modify such algorithms, even at some cost to overall predictive performance. This becomes particularly critical in resource allocation decisions, such as prioritizing patients for intensive care or specialized treatments, where algorithmic bias could disproportionately affect already vulnerable populations.

In employment contexts, algorithmic decision-making increasingly influences hiring, promotion, and compensation decisions. Our results about the relationship between accuracy loss tolerance and impact disparity have direct implications for how organizations design and deploy such systems. For instance, resume screening algorithms that show systematic bias against certain demographic groups may face growing pressure for adjustment, even if such modifications reduce overall prediction accuracy for job performance.

However, the acceptable threshold for accuracy-fairness trade-offs likely varies not only across domains but also across stakeholder groups. Our results reveal important demographic and ideological differences in these preferences. Women and politically left-leaning individuals show stronger preferences for equal impact algorithms, while men and politically right-leaning individuals place greater emphasis on accuracy maximization. These systematic differences compound the challenges institutions face in deploying algorithmic systems across different contexts. A healthcare provider serving diverse communities, for instance, may need to weigh different patient populations’ varying preferences regarding fairness versus accuracy. Similarly, employers must consider how their workforce’s demographic composition might influence reactions to algorithmic hiring or promotion systems. At a minimum, institutions should consider these differing perspectives when setting fairness thresholds and developing communication strategies around algorithmic decision-making.

This research has some limitations. As hypothetical studies, the designs are vulnerable to participant over or under-estimation. Follow-up work should validate whether stated preferences match actual choices. Field experiments debriefing people after interacting with biased algorithms would bolster ecological validity [58]. Still, our controlled experiments offer initial insight into a complex issue. Relatedly, while our studies provide insights into general public perceptions, an important question remains about how views might differ among populations directly affected by algorithmic decisions. For instance, individuals who have applied for loans, particularly those from historically disadvantaged groups, may weigh accuracy-fairness trade-offs differently based on their personal experiences with these systems. Our findings that women showed stronger preferences for equal impact algorithms suggest that group membership influences these preferences, but direct experience with algorithmic decisions might matter even more. Future research could compare algorithm preferences between the general public and specific populations - such as recent loan applicants, small business owners, or residents of areas with limited access to traditional banking. Such comparisons could reveal whether direct experience with algorithmic decisions leads to different evaluations of accuracy-fairness trade-offs. This work could also examine how personal experiences of algorithmic bias might affect trust in and acceptance of algorithmic decision-making systems.

Another important limitation is our study’s lack of geographic analysis. Given the documented relationship between geography, political ideology, and socioeconomic factors in the United States, regional variations might influence algorithmic fairness preferences. For instance, urban-rural differences in exposure to algorithmic systems, or regional variations in political attitudes toward regulation, could affect how people evaluate accuracy-fairness trade-offs. Additionally, local economic conditions and industry concentrations might shape how different communities weigh the practical implications of algorithmic decisions. Future research could explore how geographic factors - including urbanization levels, regional economic conditions, and local political climates - influence preferences for algorithmic fairness. Such analysis could help institutions better tailor their algorithmic deployment strategies to local contexts and concerns.

In conclusion, despite increasing demand for fairer algorithms, disagreements remain over implementation. Our findings imply that achieving sociotechnical alignment requires addressing conflicting human values, not just engineering solutions. Procedural fairness around standard-setting may be as critical as technical fairness [59]. More interdisciplinary research engaging social and computing sciences is essential for responsible AI governance moving forward.

## Support information

S1 File AI_fairness_SM.pdf (PDF)
