# Supplementary material for: Public perception of accuracy-fairness trade-offs in algorithmic decisions in the United States
Source: PLoS One. 2025 Mar 13;20(3):e0319861. doi: 10.1371/journal.pone.0319861 (PMC11906050; doi:10.1371/journal.pone.0319861)

Supplementary Materials

Table of Contents

Scenario Descriptions – Study 1 3
Conjoint Attributes and Attribute Levels – Study 1 4
Scenario Description – Study 2 5
Choice Tasks – Study 2 6
Measures – Studies 1 and 2 10
Table S1. Frequency of Conjoint Attributes 11
Table S2A. Marginal Means – Healthcare Scenario 12
Table 2B. Marginal Means – Lending Scenario 13
Table 2C. Marginal Means – Insurance Scenario 14
Table S3A. Difference in Marginal Means – Lending - Healthcare 15
Table S3B. Difference in Marginal Means – Insurance - Healthcare 16
Table S4A. AMCE – Healthcare Scenario 17
Table S4B. AMCE – Lending Scenario 18
Table S4C. AMCE – Insurance Scenario 19
Table 5A. Difference in AMCE – Lending – Healthcare 20
Table 5B. Difference in AMCE – Insurance – Healthcare 21
Table S6A. Marginal Means – Democrats 22
Table S6B. Marginal Means – Independents 23
Table S6C. Marginal Means – Republicans 24
Table S7A. Difference in Marginal Means – Independents - Democrats 25
Table S7B. Difference in Marginal Means – Republicans - Democrats 26
Table S8A. AMCE – Democrats 27
Table S8B. AMCE – Independents 28
Table S8C. AMCE – Republicans 29
Table S9A. Difference in AMCE – Independents - Democrats 30
Table S9B. Difference in AMCE – Republicans - Democrats 31
Figure S1. Subgroup Analysis – Equality Orientation 32
Table S10A. Marginal Means – Low Equality Orientation 33

Table S10B. Marginal Means – High Equality Orientation 34
Table S11. Difference in Marginal Means – High - Low Equality Orientation 35
Table S12A. AMCE – Low Equality Orientation 36
Table S12B. AMCE – High Equality Orientation 37
Table S13. Difference in AMCE – High - Low Equality Orientation 38
Figure S2. Subgroup Analysis Equity Orientation 39
Table S14A. Marginal Means – Low Equity Orientation 40
Table S14B. Marginal Means – High Equity Orientation 41
Table S15. Difference in Marginal Means – High - Low Equity Orientation 42
Table S16A. AMCE – Low Equity Orientation 43
Table S16B. AMCE – High Equity Orientation 44
Table S17. Difference in AMCE – High - Low Equity Orientation 45
Figure S3. Subgroup Analysis Need Orientation 46
Table S18A. Marginal Means – Low Need Orientation 47
Table S18B. Marginal Means – High Need Orientation 48
Table S19. Difference in Marginal Means – High - Low Need Orientation 49
Table S20A. AMCE – Low Need Orientation 50
Table S20B. AMCE – High Need Orientation 51
Table S21. Difference in AMCE – High - Low Need Orientation 52
Figure S4. Subgroup Analysis Entitlement Orientation 53
Table S22A. Marginal Means – Low Entitlement Orientation 54
Table S22B. Marginal Means – High Entitlement Orientation 55
Table S23. Difference in Marginal Means – High - Low Entitlement Orientation 56
Table S24A. AMCE – Low Entitlement Orientation 57
Table S24B. AMCE – High Entitlement Orientation 58
Table S25. Difference in AMCE – High - Low Entitlement Orientation 59
Table S26. Fixed effects for the choice of EIA – Study 2 full data 60
Figure S5. Effects of accuracy loss and impact disparity on choice of the EIA – Study 2 full data 61
Figure S6. Probability of Choosing the EIA by Individual Characteristics – Study 2 62

Scenario Descriptions – Study 1

Healthcare Scenario

A hospital must choose one of two data-driven algorithms to diagnose a particular disease and decide whether a patient will get a medication. Both algorithms process data about patients' symptoms and medical history to predict the presence or absence of the disease.

The Equal Treatment Algorithm has the highest overall accuracy and ignores a patient's demographic information such as gender, race, and ethnicity. However, since the data used to build the algorithm is historically biased, the algorithm can be less accurate for specific groups (i.e., members of a disadvantaged group are more likely to be misdiagnosed as not needing the medication, when in fact they do need it).

The Equal Impact Algorithm corrects for demographic biases by making small changes to the algorithm to make sure that the algorithm has the same levels of accuracy regardless of a person's demographic group. That is, a patient who has the disease has the same chance of being diagnosed as needing the medication and getting it regardless of their group membership. However, this algorithm is generally less accurate overall than the Equal Treatment Algorithm.

Lending Scenario

A bank must choose one of two data-driven algorithms for approving applications for a particular type of loan. Both algorithms process thousands of pieces of data from many different sources about people's behavior and personal characteristics to predict an applicant's likelihood of repaying a loan.

The Equal Treatment Algorithm has the highest overall accuracy and ignores an applicant's demographic information such as gender, race, and ethnicity. However, since the data used to build the algorithm is historically biased, the algorithm can be less accurate for specific groups (i.e., qualified members of a disadvantaged group are more likely to be misclassified as unlikely to repay and are thus denied a loan).

The Equal Impact Algorithm corrects for demographic biases by making small changes to the algorithm to make sure that the algorithm has the same levels of accuracy regardless of a person's demographic group. That is, a qualified applicant has the same chance of being classified as likely to repay the loan, and of receiving a loan regardless of their group membership. However, this algorithm is generally less accurate overall than the Equal Treatment Algorithm.

Insurance Scenario

An insurance company must choose one of two data-driven algorithms to assess risk and decide whether a client will get a lower price for a particular type of insurance. Both algorithms make predictions based on data they have collected about thousands of other people.

The Equal Treatment Algorithm has the highest overall accuracy and ignores a client's demographic information such as gender, race, and ethnicity. However, since the data used to build the algorithm is historically biased, the algorithm can be less accurate for specific groups (i.e., members of a disadvantaged group are more likely to be misclassified as high-risk and are thus offered a higher price).

The Equal Impact Algorithm corrects for demographic biases by making small changes to the algorithm to make sure that the risk assessment is the same regardless of a person's demographic group. That is, a truly low-risk client has the same chance of being classified as low-risk and receiving a lower price regardless of their group membership. However, this algorithm is generally less accurate overall than the Equal Treatment Algorithm.

Conjoint Attributes and Attribute Levels – Study 1

Attribute	Attribute Levels
Equal Treatment Algorithm Accuracy	The Equal Treatment Algorithm has an Overall Accuracy of **90%**.<br>[**70%**], [**80%**]
Equal Impact Algorithm Accuracy	The Equal Impact Algorithm has an Overall Accuracy of **85%**.<br>[**50%**]**,** [**60%**], [**65%**], [**70%**], [**75%**], [**80%**]
Group Disadvantaged by the Equal Treatment Algorithm	The Equal Treatment Algorithm disadvantages **women**.<br>[**People with Disabilities**], [**People of Color and Ethnic Minorities**]
Degree of Disadvantage	Under the Equal Treatment Algorithm, [disadvantaged group] are **20%** more likely to be misclassified than [regular group].<br>[**5%**], [**10%**]
Severity of Consequences (Healthcare)	If untreated, the disease usually causes **severe symptoms (e.g., heart failure, stroke, compromised immune system)**<br>[If untreated, the disease usually causes **moderate symptoms (e.g., nausea, fatigue, headache, excessive sweating)**]
Severity of Consequences (Lending)	If denied this type of loan, customers usually face **severe financial difficulties (e.g., foreclosure, eviction, poverty)**<br>[If denied this type of loan, customers usually face **moderate financial difficulties (e.g., delayed payments, reduced savings, lower credit score)**]
Severity of Consequences (Insurance)	If offered a high premium, clients usually face **severe extra costs (e.g., lack of coverage, medical bills, bankruptcy)**<br>[If offered a high premium, clients usually face **moderate extra costs (e.g., higher monthly fees, lower disposable income, reduced savings)**]
Availability (Healthcare)	The supply of this type of loan is **abundant.** Because they are very safe, and easy to administer, the loan can be offered to many customers<br>[The supply of this type of loan is **very limited**. Because they are very risky, and costly to administer the loan cannot be offered to many customers]
Availability (Lending)	The supply of this type of loan is **abundant.** Because they are very safe, and easy to administer, the loan can be offered to many customers<br>[The supply of this type of loan is **very limited**. Because they are very risky, and costly to administer the loan cannot be offered to many customers]
Availability (Insurance)	The supply of this type of insurance is **abundant.** Because it is very cheap, and simple to calculate, the insurance coverage can be provided to many clients<br>[The supply of this type of insurance is very **limited.** Because it is very expensive, and complex to calculate, this insurance coverage cannot be provided to many client]
Current Algorithm (Healthcare)	The hospital is **currently using the Equal Impact Algorithm** and is trying to decide whether to keep using it in the future or to switch to the Equal Treatment Algorithm<br>[The hospital is **currently using the Equal Treatment Algorithm** and is trying to decide whether to keep using it in the future or to switch to the Equal Impact Algorithm]<br>[The hospital is **trying to decide** whether to use the Equal Treatment Algorithm or the Equal Impact Algorithm]
Current Algorithm (Lending)	The bank is **currently using the Equal Impact Algorithm** and is trying to decide whether to keep using it in the future or to switch to the Equal Treatment Algorithm<br>[The bank is **currently using the Equal Treatment Algorithm** and is trying to decide whether to keep using it in the future or to switch to the Equal Impact Algorithm]<br>[The bank is **currently trying to decide** whether to use the Equal Treatment Algorithm or the Equal Impact Algorithm]
Current Algorithm (Insurance)	The insurance company is **currently using the Equal Impact Algorithm** and is trying to decide whether to keep using it in the future or to switch to the Equal Treatment Algorithm<br>[The insurance company is **currently using the Equal Treatment Algorithm** and is trying to decide whether to keep using it in the future or to switch to the Equal Impact Algorithm]<br>[The insurance company is **trying to decide** whether to use the Equal Treatment Algorithm or the Equal Impact Algorithm]

Scenario Description – Study 2

A bank must choose one of two data-driven algorithms for approving applications for a particular type of loan. Both algorithms process thousands of pieces of data from many different sources about people's behavior and personal characteristics to predict an applicant's likelihood of repaying a loan.

The Equal Treatment Algorithm excludes protected attributes (e.g., gender, race, and ethnicity) from the statistical model, typically resulting in higher overall accuracy. However, since the data used to build the algorithm is historically biased, it tends to have an unequal impact across demographic groups. In particular, qualified women tend to be misclassified as unlikely to repay more often than qualified men and are thus more likely to be wrongly denied a loan.

The Equal Impact Algorithm corrects for demographic biases by making small changes to the algorithm to ensure that it has the same impact on all qualified individuals regardless of their demographic group. That is, a qualified applicant has the same chance of being classified as likely to repay the loan, and of receiving the loan regardless of their group membership. However, this algorithm is generally less accurate overall than the Equal Treatment Algorithm.

In both cases, overall accuracy refers to the percentage of correct decisions (both correct approvals of qualified applicants and correct denials of unqualified applicants). Impact disparity refers to any difference in the rate of wrong denials of qualified men and qualified women.

For the bank, loan approval accuracy is closely tied to its financial performance. Higher accuracy leads to higher loan repayment rates and profitability. At the same time, achieving impact parity across demographic groups can affect the bank's reputation and long-term customer relationships.

Being wrongly denied a loan can have significant negative consequences for individuals, potentially affecting their ability to buy homes, start businesses, or invest in education.

Accuracy Loss 2% - Impact Disparity 4%

Equal Treatment Algorithm:
- This algorithm correctly classifies 90 out of each100 applicants (**90% overall accuracy**).
- Out of each 100 qualified men, 13 are wrongly denied loans (**13% wrong denials**).
- Out of each 100 qualified women, 17 are wrongly denied loans (**17% wrong denials**).

Equal Impact Algorithm:
- This algorithm correctly classifies 88 out of each 100 applicants (**88% overall accuracy**).
- Out of each 100 qualified men, 15 are wrongly denied loans (**15% wrong denials**).
- Out of each 100 qualified women, 15 are wrongly denied loans (**15% wrong denials**).

Accuracy Loss 5% - Impact Disparity 4%

Equal Treatment Algorithm:
- This algorithm correctly classifies 90 out of each100 applicants (**90% overall accuracy**).
- Out of each 100 qualified men, 13 are wrongly denied loans (**13% wrong denials**).
- Out of each 100 qualified women, 17 are wrongly denied loans (**17% wrong denials**).

Equal Impact Algorithm:
- This algorithm correctly classifies 85 out of each 100 applicants (**85% overall accuracy**).
- Out of each 100 qualified men, 15 are wrongly denied loans (**15% wrong denials**).
- Out of each 100 qualified women, 15 are wrongly denied loans (**15% wrong denials**).

Accuracy Loss 10% - Impact Disparity 4%

Equal Treatment Algorithm:
- This algorithm correctly classifies 90 out of each100 applicants (**90% overall accuracy**).
- Out of each 100 qualified men, 13 are wrongly denied loans (**13% wrong denials**).
- Out of each 100 qualified women, 17 are wrongly denied loans (**17% wrong denials**).

Equal Impact Algorithm:
- This algorithm correctly classifies 80 out of each 100 applicants (**80% overall accuracy**).
- Out of each 100 qualified men, 15 are wrongly denied loans (**15% wrong denials**).
- Out of each 100 qualified women, 15 are wrongly denied loans (**15% wrong denials**).

Accuracy Loss 15% - Impact Disparity 4%

Equal Treatment Algorithm:
- This algorithm correctly classifies 90 out of each100 applicants (**90% overall accuracy**).
- Out of each 100 qualified men, 13 are wrongly denied loans (**13% wrong denials**).
- Out of each 100 qualified women, 17 are wrongly denied loans (**17% wrong denials**).

Equal Impact Algorithm:
- This algorithm correctly classifies 75 out of each 100 applicants (**75% overall accuracy**).
- Out of each 100 qualified men, 15 are wrongly denied loans (**15% wrong denials**).
- Out of each 100 qualified women, 15 are wrongly denied loans (**15% wrong denials**).

Accuracy Loss 20% - Impact Disparity 4%

Equal Treatment Algorithm:
- This algorithm correctly classifies 90 out of each100 applicants (**90% overall accuracy**).
- Out of each 100 qualified men, 13 are wrongly denied loans (**13% wrong denials**).
- Out of each 100 qualified women, 17 are wrongly denied loans (**17% wrong denials**).

Equal Impact Algorithm:
- This algorithm correctly classifies 70 out of each 100 applicants (**70% overall accuracy**).
- Out of each 100 qualified men, 15 are wrongly denied loans (**15% wrong denials**).
- Out of each 100 qualified women, 15 are wrongly denied loans (**15% wrong denials**).

Accuracy Loss 2% - Impact Disparity 10%

Equal Treatment Algorithm:
- This algorithm correctly classifies 90 out of each100 applicants (**90% overall accuracy**).
- Out of each 100 qualified men, 10 are wrongly denied loans (**10% wrong denials**).
- Out of each 100 qualified women, 20 are wrongly denied loans (**20% wrong denials**).

Equal Impact Algorithm:
- This algorithm correctly classifies 88 out of each 100 applicants (**88% overall accuracy**).
- Out of each 100 qualified men, 15 are wrongly denied loans (**15% wrong denials**).
- Out of each 100 qualified women, 15 are wrongly denied loans (**15% wrong denials**).

Accuracy Loss 5% - Impact Disparity 10%

Equal Treatment Algorithm:
- This algorithm correctly classifies 90 out of each100 applicants (**90% overall accuracy**).
- Out of each 100 qualified men, 10 are wrongly denied loans (**10% wrong denials**).
- Out of each 100 qualified women, 20 are wrongly denied loans (**20% wrong denials**).

Equal Impact Algorithm:
- This algorithm correctly classifies 85 out of each 100 applicants (**85% overall accuracy**).
- Out of each 100 qualified men, 15 are wrongly denied loans (**15% wrong denials**).
- Out of each 100 qualified women, 15 are wrongly denied loans (**15% wrong denials**).

Accuracy Loss 10% - Impact Disparity 10%

Equal Treatment Algorithm:
- This algorithm correctly classifies 90 out of each100 applicants (**90% overall accuracy**).
- Out of each 100 qualified men, 10 are wrongly denied loans (**10% wrong denials**).
- Out of each 100 qualified women, 20 are wrongly denied loans (**20% wrong denials**).

Equal Impact Algorithm:
- This algorithm correctly classifies 80 out of each 100 applicants (**80% overall accuracy**).
- Out of each 100 qualified men, 15 are wrongly denied loans (**15% wrong denials**).
- Out of each 100 qualified women, 15 are wrongly denied loans (**15% wrong denials**).

Accuracy Loss 15% - Impact Disparity 10%

Equal Treatment Algorithm:

- This algorithm correctly classifies 90 out of each100 applicants (**90% overall accuracy**).
- Out of each 100 qualified men, 10 are wrongly denied loans (**10% wrong denials**).
- Out of each 100 qualified women, 20 are wrongly denied loans (**20% wrong denials**).

Equal Impact Algorithm:

- This algorithm correctly classifies 75 out of each 100 applicants (**75% overall accuracy**).
- Out of each 100 qualified men, 15 are wrongly denied loans (**15% wrong denials**).
- Out of each 100 qualified women, 15 are wrongly denied loans (**15% wrong denials**).

Accuracy Loss 20% - Impact Disparity 10%

Equal Treatment Algorithm:

- This algorithm correctly classifies 90 out of each100 applicants (**90% overall accuracy**).
- Out of each 100 qualified men, 10 are wrongly denied loans (**10% wrong denials**).
- Out of each 100 qualified women, 20 are wrongly denied loans (**20% wrong denials**).

Equal Impact Algorithm:

- This algorithm correctly classifies 70 out of each 100 applicants (**70% overall accuracy**).
- Out of each 100 qualified men, 15 are wrongly denied loans (**15% wrong denials**).
- Out of each 100 qualified women, 15 are wrongly denied loans (**15% wrong denials**).

Accuracy Loss 2% - Impact Disparity 20%

Equal Treatment Algorithm:

- This algorithm correctly classifies 90 out of each100 applicants (**90% overall accuracy**).
- Out of each 100 qualified men, 5 are wrongly denied loans (**5% wrong denials**).
- Out of each 100 qualified women, 25 are wrongly denied loans (**25% wrong denials**).

Equal Impact Algorithm:

- This algorithm correctly classifies 88 out of each 100 applicants (**88% overall accuracy**).
- Out of each 100 qualified men, 15 are wrongly denied loans (**15% wrong denials**).
- Out of each 100 qualified women, 15 are wrongly denied loans (**15% wrong denials**).

Accuracy Loss 5% - Impact Disparity 20%

Equal Treatment Algorithm:

- This algorithm correctly classifies 90 out of each100 applicants (**90% overall accuracy**).
- Out of each 100 qualified men, 5 are wrongly denied loans (**5% wrong denials**).
- Out of each 100 qualified women, 25 are wrongly denied loans (**25% wrong denials**).

Equal Impact Algorithm:

- This algorithm correctly classifies 85 out of each 100 applicants (**85% overall accuracy**).
- Out of each 100 qualified men, 15 are wrongly denied loans (**15% wrong denials**).
- Out of each 100 qualified women, 15 are wrongly denied loans (**15% wrong denials**).

<u>Accuracy Loss 10% - Impact Disparity 20%</u>

Equal Treatment Algorithm:
- This algorithm correctly classifies 90 out of each100 applicants (**90% overall accuracy**).
- Out of each 100 qualified men, 5 are wrongly denied loans (**5% wrong denials**).
- Out of each 100 qualified women, 25 are wrongly denied loans (**25% wrong denials**).

Equal Impact Algorithm:
- This algorithm correctly classifies 80 out of each 100 applicants (**80% overall accuracy**).
- Out of each 100 qualified men, 15 are wrongly denied loans (**15% wrong denials**).
- Out of each 100 qualified women, 15 are wrongly denied loans (**15% wrong denials**).

<u>Accuracy Loss 15% - Impact Disparity 20%</u>

Equal Treatment Algorithm:
- This algorithm correctly classifies 90 out of each100 applicants (**90% overall accuracy**).
- Out of each 100 qualified men, 5 are wrongly denied loans (**5% wrong denials**).
- Out of each 100 qualified women, 25 are wrongly denied loans (**25% wrong denials**).

Equal Impact Algorithm:
- This algorithm correctly classifies 75 out of each 100 applicants (**75% overall accuracy**).
- Out of each 100 qualified men, 15 are wrongly denied loans (**15% wrong denials**).
- Out of each 100 qualified women, 15 are wrongly denied loans (**15% wrong denials**).

<u>Accuracy Loss 20% - Impact Disparity 20%</u>

Equal Treatment Algorithm:
- This algorithm correctly classifies 90 out of each100 applicants (**90% overall accuracy**).
- Out of each 100 qualified men, 5 are wrongly denied loans (**5% wrong denials**).
- Out of each 100 qualified women, 25 are wrongly denied loans (**25% wrong denials**).

Equal Impact Algorithm:
- This algorithm correctly classifies 70 out of each 100 applicants (**70% overall accuracy**).
- Out of each 100 qualified men, 15 are wrongly denied loans (**15% wrong denials**).
- Out of each 100 qualified women, 15 are wrongly denied loans (**15% wrong denials**).

Measures – Studies 1 and 2

Attention Check

How many choices will you make in the study? (5, 12, 15, 20)

DV: Choice

Which algorithm should the company use going forward? (Equal Treatment Algorithm, Equal Impact Algorithm)

DV: Acceptability (1 = Totally unacceptable, 5 = Perfectly acceptable)

How acceptable is it for the company to use the Equal Treatment Algorithm?
How acceptable is it for the company to use the Equal Impact Algorithm?

The basic Social Justice Orientations (BSJO) Scale (1 = Strongly disagree, 5 = Strongly agree)

There are different ideas about how a society can be fair and just. What is your personal opinion about this? Please state to what extent you agree or disagree with each of the following items:

1. A society is just if all people have sufficient nutrition, shelter, clothing as well as access to education and medical care.
2. It is just if hard working people earn more than others.
3. It is just if all people have the same living conditions.
4. It is just if members of respectable families have certain advantages in their lives.
5. A society is just if it takes care of those who are poor and needy.
6. It is just if people who have achieved good reputation and wealth profit from this later in life.
7. A society is just if there are only minor income disparities between people.
8. A society is just if differences in income and assets reflect performance differences between people.
9. It is just if every person receives only that which has been acquired through their own efforts.
10. It is just if people taking care of their children or their dependent relatives receive special support and benefits.
11. It is just if income and wealth are equally distributed among the members of our society.
12. It is fair if people on a higher level of society have better living conditions than those on the lower level.

Equality (items 3, 7, 11); Need (items 1, 5, 10); Equity (items 2, 8, 9), Entitlement (items 4, 6, 12)

Political Affiliation

Which federal political party do you most identify with? (Democrat, Republican, Independent, Other)

Gender

Please indicate your gender (Male, Female, Non-Binary, Prefer not to answer)

Age

What is your age?

Ethnicity

Please select all race(s) and/or ethnicities you identify as (American Indian or Alaska Native, Asian, Black or African American, Hispanic or Latino or Spanish Origin of any race, Native Hawaiian or Other Pacific Islander, White, Other)

Disability Status

Do you have a disability? (Yes, No, Prefer not to answer)

Table S1. Frequency of Conjoint Attributes

Attribute	Level	Frequency	Percent
ETA Accuracy	70%	7,891	33.3%
	80%	7,882	33.3%
	90%	7,927	33.4%
Accuracy Loss with EIA	5%	7,881	33.3%
	10%	7,870	33.2%
	20%	7,949	33.5%
Group Disadvantaged by the ETA	Women	7,930	33.5%
	POC	7,843	33.1%
	PWD	7,924	33.4%
Degree of Disadvantage	5%	7,791	32.9%
	10%	8,010	33.8%
	20%	7,899	33.3%
Severity of Consequences	Moderate	11,923	50.3%
	Severe	11,777	49.7%
Availability	High	11,675	49.3%
	Low	12,025	50.7%
Current Algorithm	None	7,869	33.2%
	ETA	7,884	33.3%
	EIA	7,947	33.5%

Table S2A. Marginal Means – Healthcare Scenario

Attribute	Level	Estimate	SE	Lower CI	Upper CI
ETA Accuracy	70%	0.50	0.01	0.48	0.53
ETA Accuracy	80%	0.53	0.01	0.51	0.56
ETA Accuracy	90%	0.52	0.01	0.50	0.54
Accuracy Loss	5%	0.63	0.01	0.61	0.66
Accuracy Loss	10%	0.54	0.01	0.52	0.57
Accuracy Loss	20%	0.38	0.01	0.36	0.40
Disadvantaged Group	Women	0.51	0.01	0.49	0.53
Disadvantaged Group	POC	0.55	0.01	0.53	0.57
Disadvantaged Group	PWD	0.50	0.01	0.48	0.52
Magnitude	5%	0.45	0.01	0.42	0.47
Magnitude	10%	0.50	0.01	0.48	0.53
Magnitude	20%	0.60	0.01	0.58	0.62
Consequence	Moderate	0.50	0.01	0.48	0.52
Consequence	Severe	0.53	0.01	0.51	0.55
Availability	High	0.52	0.01	0.51	0.54
Availability	Low	0.51	0.01	0.49	0.53
Default	None	0.52	0.01	0.50	0.55
Default	ETA	0.50	0.01	0.48	0.52
Default	EIA	0.54	0.01	0.51	0.56

Table 2B. Marginal Means – Lending Scenario

Attribute	Level	Estimate	SE	Lower CI	Upper CI
ETA Accuracy	70%	0.53	0.01	0.50	0.55
ETA Accuracy	80%	0.55	0.01	0.53	0.57
ETA Accuracy	90%	0.57	0.01	0.55	0.59
Accuracy Loss	5%	0.67	0.01	0.65	0.69
Accuracy Loss	10%	0.57	0.01	0.55	0.59
Accuracy Loss	20%	0.41	0.01	0.38	0.43
Disadvantaged Group	Women	0.54	0.01	0.51	0.56
Disadvantaged Group	POC	0.59	0.01	0.56	0.61
Disadvantaged Group	PWD	0.52	0.01	0.50	0.54
Magnitude	5%	0.48	0.01	0.46	0.50
Magnitude	10%	0.55	0.01	0.53	0.57
Magnitude	20%	0.61	0.01	0.59	0.64
Consequence	Moderate	0.53	0.01	0.51	0.55
Consequence	Severe	0.57	0.01	0.55	0.59
Availability	High	0.55	0.01	0.53	0.57
Availability	Low	0.55	0.01	0.53	0.57
Default	None	0.55	0.01	0.53	0.57
Default	ETA	0.53	0.01	0.51	0.56
Default	EIA	0.56	0.01	0.54	0.58

Table 2C. Marginal Means – Insurance Scenario

Attribute	Level	Estimate	SE	Lower CI	Upper CI
ETA Accuracy	70%	0.51	0.01	0.49	0.54
ETA Accuracy	80%	0.55	0.01	0.53	0.58
ETA Accuracy	90%	0.55	0.01	0.52	0.57
Accuracy Loss	5%	0.64	0.01	0.62	0.66
Accuracy Loss	10%	0.58	0.01	0.56	0.60
Accuracy Loss	20%	0.40	0.01	0.38	0.42
Disadvantaged Group	Women	0.54	0.01	0.52	0.56
Disadvantaged Group	POC	0.55	0.01	0.53	0.58
Disadvantaged Group	PWD	0.52	0.01	0.50	0.55
Magnitude	5%	0.47	0.01	0.45	0.50
Magnitude	10%	0.53	0.01	0.51	0.56
Magnitude	20%	0.61	0.01	0.59	0.63
Consequence	Moderate	0.53	0.01	0.51	0.55
Consequence	Severe	0.55	0.01	0.53	0.57
Availability	High	0.53	0.01	0.51	0.55
Availability	Low	0.54	0.01	0.52	0.56
Default	None	0.53	0.01	0.51	0.55
Default	ETA	0.53	0.01	0.51	0.56
Default	EIA	0.55	0.01	0.53	0.57

Table S3A. Difference in Marginal Means – Lending - Healthcare

Attribute	Level	Estimate	SE	*z*	*p*	Lower CI	Upper CI
ETA Accuracy	70%	0.02	0.02	1.38	0.17	-0.01	0.05
ETA Accuracy	80%	0.01	0.02	0.92	0.36	-0.02	0.05
ETA Accuracy	90%	0.05	0.02	3.12	0.00	0.02	0.08
Accuracy Loss	5%	0.04	0.02	2.47	0.01	0.01	0.07
Accuracy Loss	10%	0.02	0.02	1.49	0.14	-0.01	0.06
Accuracy Loss	20%	0.03	0.02	1.77	0.08	0.00	0.06
Disadvantaged Group	Women	0.03	0.02	1.67	0.09	0.00	0.06
Disadvantaged Group	POC	0.04	0.02	2.26	0.02	0.00	0.07
Disadvantaged Group	PWD	0.02	0.02	1.44	0.15	-0.01	0.05
Magnitude	5%	0.03	0.02	2.08	0.04	0.00	0.07
Magnitude	10%	0.05	0.02	2.82	<0.01	0.01	0.08
Magnitude	20%	0.01	0.02	0.72	0.47	-0.02	0.04
Consequence	Moderate	0.02	0.01	1.62	0.11	0.00	0.05
Consequence	Severe	0.04	0.01	2.47	0.01	0.01	0.06
Availability	High	0.02	0.01	1.59	0.11	-0.01	0.05
Availability	Low	0.03	0.01	2.49	0.01	0.01	0.06
Default	None	0.02	0.02	1.51	0.13	-0.01	0.06
Default	ETA	0.04	0.02	2.22	0.03	0.00	0.07
Default	EIA	0.03	0.02	1.64	0.10	-0.01	0.06

Table S3B. Difference in Marginal Means – Insurance - Healthcare

Attribute	Level	Estimate	SE	*z*	*p*	Lower CI	Upper CI
ETA Accuracy	70%	0.01	0.02	0.62	0.53	-0.02	0.04
ETA Accuracy	80%	0.02	0.02	1.31	0.19	-0.01	0.05
ETA Accuracy	90%	0.03	0.02	1.67	0.09	0.00	0.06
Accuracy Loss	5%	0.00	0.02	0.29	0.77	-0.03	0.04
Accuracy Loss	10%	0.04	0.02	2.26	0.02	0.00	0.07
Accuracy Loss	20%	0.02	0.02	1.26	0.21	-0.01	0.05
Disadvantaged Group	Women	0.03	0.02	1.73	0.08	0.00	0.06
Disadvantaged Group	POC	0.00	0.02	0.28	0.78	-0.03	0.04
Disadvantaged Group	PWD	0.03	0.02	1.56	0.12	-0.01	0.06
Magnitude	5%	0.03	0.02	1.57	0.12	-0.01	0.06
Magnitude	10%	0.03	0.02	1.79	0.07	0.00	0.06
Magnitude	20%	0.01	0.02	0.46	0.64	-0.02	0.04
Consequence	Moderate	0.02	0.01	1.74	0.08	0.00	0.05
Consequence	Severe	0.01	0.01	1.02	0.31	-0.01	0.04
Availability	High	0.01	0.01	0.69	0.49	-0.02	0.04
Availability	Low	0.03	0.01	2.06	0.04	0.00	0.06
Default	None	0.01	0.02	0.37	0.71	-0.03	0.04
Default	ETA	0.04	0.02	2.24	0.02	0.00	0.07
Default	EIA	0.02	0.02	0.98	0.32	-0.02	0.05

Table S4A. AMCE – Healthcare Scenario

**Attribute**	**Level**	**Estimate**	**SE**	***z***	***p***	**Lower CI**	**Upper CI**
ETA Accuracy	70%	0.00					
ETA Accuracy	80%	0.03	0.01	2.39	0.02	0.01	0.06
ETA Accuracy	90%	0.02	0.01	1.15	0.25	-0.01	0.04
Accuracy Loss	5%	0.00					
Accuracy Loss	10%	-0.09	0.01	-6.56	<0.01	-0.12	-0.06
Accuracy Loss	20%	-0.26	0.01	-18.05	<0.01	-0.29	-0.23
Disadvantaged Group	Women	0.00					
Disadvantaged Group	POC	0.04	0.01	3.15	<0.01	0.02	0.07
Disadvantaged Group	PWD	-0.01	0.01	-0.46	0.65	-0.03	0.02
Magnitude	5%	0.00					
Magnitude	10%	0.05	0.01	3.98	<0.01	0.03	0.08
Magnitude	20%	0.16	0.01	11.17	<0.01	0.13	0.18
Consequence	Moderate	0.00					
Consequence	Severe	0.03	0.01	2.96	<0.01	0.01	0.06
Availability	High	0.00					
Availability	Low	-0.01	0.01	-0.51	0.61	-0.03	0.02
Default	None	0.00					
Default	ETA	-0.02	0.01	-1.82	0.07	-0.05	0.00
Default	EIA	0.01	0.01	0.94	0.34	-0.01	0.04

Table S4B. AMCE – Lending Scenario

**Attribute**	**Level**	**Estimate**	**SE**	***z***	***p***	**Lower CI**	**Upper CI**
ETA Accuracy	70%	0.00					
ETA Accuracy	80%	0.02	0.01	1.41	0.16	-0.01	0.04
ETA Accuracy	90%	0.04	0.01	3.02	0.00	0.01	0.07
Accuracy Loss	5%	0.00					
Accuracy Loss	10%	-0.10	0.01	-7.51	<0.01	-0.13	-0.07
Accuracy Loss	20%	-0.26	0.01	-18.57	<0.01	-0.29	-0.23
Disadvantaged Group	Women	0.00					
Disadvantaged Group	POC	0.05	0.01	3.72	<0.01	0.02	0.08
Disadvantaged Group	PWD	-0.01	0.01	-0.55	0.58	-0.03	0.02
Magnitude	5%	0.00					
Magnitude	10%	0.07	0.01	5.18	<0.01	0.04	0.09
Magnitude	20%	0.13	0.01	9.51	<0.01	0.10	0.16
Consequence	Moderate	0.00					
Consequence	Severe	0.04	0.01	3.27	<0.01	0.01	0.06
Availability	High	0.00					
Availability	Low	0.00	0.01	0.22	0.82	-0.02	0.02
Default	None	0.00					
Default	ETA	-0.01	0.01	-0.83	0.41	-0.04	0.01
Default	EIA	0.02	0.01	1.20	0.23	-0.01	0.04

Table S4C. AMCE – Insurance Scenario

Attribute	Level	Estimate	SE	*z*	*p*	Lower CI	Upper CI
ETA Accuracy	70%	0.00					
ETA Accuracy	80%	0.04	0.01	2.83	<0.01	0.01	0.06
ETA Accuracy	90%	0.02	0.01	1.74	0.08	0.00	0.05
Accuracy Loss	5%	0.00					
Accuracy Loss	10%	-0.06	0.01	-4.32	<0.01	-0.09	-0.03
Accuracy Loss	20%	-0.24	0.01	-16.74	<0.01	-0.27	-0.21
Disadvantaged Group	Women	0.00					
Disadvantaged Group	POC	0.02	0.01	1.55	0.12	-0.01	0.05
Disadvantaged Group	PWD	-0.01	0.01	-0.80	0.42	-0.04	0.02
Magnitude	5%	0.00					
Magnitude	10%	0.06	0.01	4.15	<0.01	0.03	0.08
Magnitude	20%	0.13	0.01	9.36	<0.01	0.11	0.16
Consequence	Moderate	0.00					
Consequence	Severe	0.02	0.01	2.17	0.03	0.00	0.05
Availability	High	0.00					
Availability	Low	0.01	0.01	0.76	0.44	-0.01	0.03
Default	None	0.00					
Default	ETA	0.00	0.01	0.12	0.90	-0.02	0.03
Default	EIA	0.02	0.01	1.30	0.19	-0.01	0.04

Table 5A. Difference in AMCE – Lending – Healthcare

**Attribute**	**Level**	**Estimate**	**SE**	***z***	***p***	**Lower CI**	**Upper CI**
ETA Accuracy	80%	-0.01	0.02	-0.73	0.47	-0.05	0.02
ETA Accuracy	90%	0.03	0.02	1.36	0.17	-0.01	0.06
Accuracy Loss	10%	-0.01	0.02	-0.59	0.55	-0.05	0.03
Accuracy Loss	20%	0.00	0.02	-0.18	0.86	-0.04	0.03
Disadvantaged Group	POC	0.01	0.02	0.43	0.67	-0.03	0.04
Disadvantaged Group	PWD	0.00	0.02	-0.07	0.94	-0.04	0.04
Magnitude	10%	0.01	0.02	0.80	0.42	-0.02	0.05
Magnitude	20%	-0.03	0.02	-1.38	0.17	-0.06	0.01
Consequence	Severe	0.00	0.02	0.16	0.87	-0.03	0.03
Availability	Low	0.01	0.02	0.53	0.60	-0.02	0.04
Default	ETA	0.01	0.02	0.72	0.47	-0.02	0.05
Default	EIA	0.00	0.02	0.20	0.84	-0.03	0.04

Table 5B. Difference in AMCE – Insurance – Healthcare

**Attribute**	**Level**	**Estimate**	**SE**	***z***	***p***	**Lower CI**	**Upper CI**
ETA Accuracy	80%	0.01	0.02	0.31	0.75	-0.03	0.04
ETA Accuracy	90%	0.01	0.02	0.45	0.65	-0.03	0.04
Accuracy Loss	10%	0.03	0.02	1.53	0.13	-0.01	0.07
Accuracy Loss	20%	0.02	0.02	0.97	0.33	-0.02	0.06
Disadvantaged Group	POC	-0.02	0.02	-1.19	0.23	-0.06	0.01
Disadvantaged Group	PWD	0.00	0.02	-0.26	0.79	-0.04	0.03
Magnitude	10%	0.00	0.02	0.16	0.87	-0.03	0.04
Magnitude	20%	-0.02	0.02	-1.24	0.22	-0.06	0.01
Consequence	Severe	-0.01	0.02	-0.60	0.55	-0.04	0.02
Availability	Low	0.01	0.02	0.90	0.37	-0.02	0.04
Default	ETA	0.03	0.02	1.37	0.17	-0.01	0.06
Default	EIA	0.00	0.02	0.25	0.80	-0.03	0.04

Table S6A. Marginal Means – Democrats

Attribute	Level	Estimate	SE	Lower CI	Upper CI
Scenario	Healthcare	0.58	0.01	0.55	0.60
Scenario	Lending	0.60	0.01	0.58	0.62
Scenario	Insurance	0.60	0.01	0.57	0.62
ETA Accuracy	70%	0.58	0.01	0.55	0.60
ETA Accuracy	80%	0.59	0.01	0.57	0.62
ETA Accuracy	90%	0.60	0.01	0.58	0.63
Accuracy Loss	5%	0.70	0.01	0.68	0.72
Accuracy Loss	10%	0.62	0.01	0.60	0.64
Accuracy Loss	20%	0.45	0.01	0.43	0.47
Disadvantaged Group	Women	0.58	0.01	0.56	0.60
Disadvantaged Group	POC	0.62	0.01	0.60	0.64
Disadvantaged Group	PWD	0.58	0.01	0.55	0.60
Magnitude	5%	0.51	0.01	0.49	0.54
Magnitude	10%	0.59	0.01	0.57	0.62
Magnitude	20%	0.67	0.01	0.64	0.69
Consequence	Moderate	0.58	0.01	0.56	0.60
Consequence	Severe	0.60	0.01	0.58	0.62
Availability	High	0.59	0.01	0.57	0.61
Availability	Low	0.59	0.01	0.57	0.61
Default	None	0.59	0.01	0.57	0.61
Default	ETA	0.58	0.01	0.56	0.60
Default	EIA	0.60	0.01	0.58	0.63

Table S6B. Marginal Means – Independents

Attribute	Level	Estimate	SE	Lower CI	Upper CI
Scenario	Healthcare	0.50	0.02	0.47	0.53
Scenario	Lending	0.54	0.02	0.51	0.57
Scenario	Insurance	0.52	0.02	0.49	0.56
ETA Accuracy	70%	0.49	0.02	0.45	0.52
ETA Accuracy	80%	0.55	0.02	0.51	0.58
ETA Accuracy	90%	0.53	0.02	0.50	0.56
Accuracy Loss	5%	0.65	0.02	0.61	0.68
Accuracy Loss	10%	0.55	0.02	0.52	0.59
Accuracy Loss	20%	0.37	0.02	0.33	0.40
Disadvantaged Group	Women	0.51	0.02	0.48	0.54
Disadvantaged Group	POC	0.56	0.02	0.52	0.59
Disadvantaged Group	PWD	0.50	0.02	0.46	0.53
Magnitude	5%	0.45	0.02	0.42	0.48
Magnitude	10%	0.50	0.02	0.47	0.54
Magnitude	20%	0.61	0.02	0.58	0.64
Consequence	Moderate	0.50	0.01	0.47	0.53
Consequence	Severe	0.54	0.02	0.51	0.57
Availability	High	0.53	0.02	0.50	0.56
Availability	Low	0.52	0.01	0.49	0.54
Default	None	0.52	0.02	0.49	0.55
Default	ETA	0.50	0.02	0.47	0.53
Default	EIA	0.54	0.02	0.50	0.57

Table S6C. Marginal Means – Republicans

Attribute	Level	Estimate	SE	Lower CI	Upper CI
Scenario	Healthcare	0.40	0.02	0.37	0.43
Scenario	Lending	0.42	0.02	0.38	0.45
Scenario	Insurance	0.41	0.02	0.38	0.45
ETA Accuracy	70%	0.41	0.02	0.37	0.44
ETA Accuracy	80%	0.41	0.02	0.38	0.45
ETA Accuracy	90%	0.41	0.02	0.37	0.45
Accuracy Loss	5%	0.50	0.02	0.46	0.55
Accuracy Loss	10%	0.43	0.02	0.40	0.47
Accuracy Loss	20%	0.29	0.02	0.26	0.33
Disadvantaged Group	Women	0.41	0.02	0.38	0.45
Disadvantaged Group	POC	0.43	0.02	0.39	0.46
Disadvantaged Group	PWD	0.39	0.02	0.36	0.43
Magnitude	5%	0.37	0.02	0.33	0.41
Magnitude	10%	0.40	0.02	0.36	0.43
Magnitude	20%	0.46	0.02	0.42	0.50
Consequence	Moderate	0.39	0.02	0.36	0.42
Consequence	Severe	0.43	0.02	0.40	0.46
Availability	High	0.40	0.02	0.37	0.44
Availability	Low	0.42	0.02	0.38	0.45
Default	None	0.41	0.02	0.37	0.44
Default	ETA	0.40	0.02	0.37	0.44
Default	EIA	0.42	0.02	0.38	0.46

Table S7A. Difference in Marginal Means – Independents - Democrats

**Attribute**	**Level**	**Estimate**	**SE**	***z***	***p***	**Lower CI**	**Upper CI**
Scenario	Healthcare	-0.08	0.02	-4.07	<0.01	-0.11	-0.04
Scenario	Lending	-0.06	0.02	-3.39	<0.01	-0.10	-0.03
Scenario	Insurance	-0.07	0.02	-3.69	<0.01	-0.11	-0.03
ETA Accuracy	70%	-0.09	0.02	-4.56	<0.01	-0.13	-0.05
ETA Accuracy	80%	-0.05	0.02	-2.48	<0.01	-0.09	-0.01
ETA Accuracy	90%	-0.07	0.02	-3.61	<0.01	-0.11	-0.03
Accuracy Loss	5%	-0.06	0.02	-2.84	<0.01	-0.10	-0.02
Accuracy Loss	10%	-0.07	0.02	-3.44	<0.01	-0.11	-0.03
Accuracy Loss	20%	-0.08	0.02	-4.09	<0.01	-0.12	-0.04
Disadvantaged Group	Women	-0.07	0.02	-3.47	<0.01	-0.11	-0.03
Disadvantaged Group	POC	-0.06	0.02	-3.18	<0.01	-0.10	-0.02
Disadvantaged Group	PWD	-0.08	0.02	-3.97	<0.01	-0.12	-0.04
Magnitude	5%	-0.06	0.02	-3.13	<0.01	-0.10	-0.02
Magnitude	10%	-0.09	0.02	-4.50	<0.01	-0.13	-0.05
Magnitude	20%	-0.06	0.02	-2.97	<0.01	-0.10	-0.02
Consequence	Moderate	-0.08	0.02	-4.42	<0.01	-0.12	-0.04
Consequence	Severe	-0.06	0.02	-3.32	<0.01	-0.10	-0.03
Availability	High	-0.07	0.02	-3.63	<0.01	-0.10	-0.03
Availability	Low	-0.07	0.02	-4.09	<0.01	-0.11	-0.04
Default	None	-0.07	0.02	-3.42	<0.01	-0.11	-0.03
Default	ETA	-0.08	0.02	-3.95	<0.01	-0.11	-0.04
Default	EIA	-0.07	0.02	-3.43	<0.01	-0.11	-0.03

Table S7B. Difference in Marginal Means – Republicans - Democrats

**Attribute**	**Level**	**Estimate**	**SE**	***z***	***p***	**Lower CI**	**Upper CI**
Scenario	Healthcare	-0.18	0.02	-8.54	<0.01	-0.22	-0.14
Scenario	Lending	-0.19	0.02	-8.73	<0.01	-0.23	-0.14
Scenario	Insurance	-0.18	0.02	-8.48	<0.01	-0.22	-0.14
ETA Accuracy	70%	-0.17	0.02	-7.73	<0.01	-0.21	-0.13
ETA Accuracy	80%	-0.18	0.02	-8.25	<0.01	-0.22	-0.14
ETA Accuracy	90%	-0.20	0.02	-8.75	<0.01	-0.24	-0.15
Accuracy Loss	5%	-0.20	0.02	-8.35	<0.01	-0.25	-0.15
Accuracy Loss	10%	-0.19	0.02	-8.18	<0.01	-0.23	-0.14
Accuracy Loss	20%	-0.16	0.02	-7.36	<0.01	-0.20	-0.11
Disadvantaged Group	Women	-0.17	0.02	-7.54	<0.01	-0.21	-0.12
Disadvantaged Group	POC	-0.19	0.02	-8.46	<0.01	-0.24	-0.15
Disadvantaged Group	PWD	-0.18	0.02	-8.42	<0.01	-0.23	-0.14
Magnitude	5%	-0.15	0.02	-6.35	<0.01	-0.19	-0.10
Magnitude	10%	-0.20	0.02	-9.08	<0.01	-0.24	-0.15
Magnitude	20%	-0.20	0.02	-9.09	<0.01	-0.25	-0.16
Consequence	Moderate	-0.19	0.02	-9.42	<0.01	-0.23	-0.15
Consequence	Severe	-0.17	0.02	-8.42	<0.01	-0.21	-0.13
Availability	High	-0.19	0.02	-9.48	<0.01	-0.23	-0.15
Availability	Low	-0.17	0.02	-8.41	<0.01	-0.21	-0.13
Default	None	-0.19	0.02	-8.51	<0.01	-0.23	-0.14
Default	ETA	-0.18	0.02	-8.06	<0.01	-0.22	-0.13
Default	EIA	-0.18	0.02	-8.28	<0.01	-0.23	-0.14

Table S8A. AMCE – Democrats

**Attribute**	**Level**	**Estimate**	**SE**	***z***	***p***	**Lower CI**	**Upper CI**
Scenario	Healthcare	0.00					
Scenario	Lending	0.03	0.01	3.06	<0.01	0.01	0.05
Scenario	Insurance	0.02	0.01	2.20	0.03	<0.01	0.04
ETA Accuracy	70%	0.00					
ETA Accuracy	80%	0.02	0.01	1.62	0.10	<0.01	0.04
ETA Accuracy	90%	0.03	0.01	2.33	0.02	<0.01	0.05
Accuracy Loss	5%	0.00					
Accuracy Loss	10%	-0.08	0.01	-7.69	<0.01	-0.10	-0.06
Accuracy Loss	20%	-0.25	0.01	-19.76	<0.01	-0.28	-0.23
Disadvantaged Group	Women	0.00			<0.01		
Disadvantaged Group	POC	0.04	0.01	4.02	<0.01	0.02	0.06
Disadvantaged Group	PWD	0.00	0.01	-0.11	0.92	-0.02	0.02
Magnitude	5%	0.00					
Magnitude	10%	0.08	0.01	6.86	<0.01	0.05	0.10
Magnitude	20%	0.15	0.01	12.07	<0.01	0.13	0.18
Consequence	Moderate	0.00					
Consequence	Severe	0.02	0.01	2.49	0.01	<0.01	0.04
Availability	High	0.00					
Availability	Low	0.00	0.01	-0.52	0.60	-0.02	0.01
Default	None	0.00					
Default	ETA	-0.01	0.01	-1.14	0.25	-0.03	0.01
Default	EIA	0.02	0.01	1.42	0.15	-0.01	0.04

Table S8B. AMCE – Independents

**Attribute**	**Level**	**Estimate**	**SE**	***z***	***p***	**Lower CI**	**Upper CI**
Scenario	Healthcare	0.00					
Scenario	Lending	0.04	0.01	3.36	<0.01	0.02	0.07
Scenario	Insurance	0.03	0.01	2.49	0.01	0.01	0.06
ETA Accuracy	70%	0.00					
ETA Accuracy	80%	0.06	0.01	4.21	<0.01	0.03	0.09
ETA Accuracy	90%	0.04	0.01	2.52	0.01	0.01	0.07
Accuracy Loss	5%	0.00					
Accuracy Loss	10%	-0.09	0.02	-5.80	<0.01	-0.12	-0.06
Accuracy Loss	20%	-0.28	0.02	-15.93	<0.01	-0.31	-0.24
Disadvantaged Group	Women	0.00					
Disadvantaged Group	POC	0.05	0.02	3.05	<0.01	0.02	0.08
Disadvantaged Group	PWD	-0.01	0.01	-0.65	0.52	-0.04	0.02
Magnitude	5%	0.00					
Magnitude	10%	0.05	0.02	3.09	<0.01	0.02	0.08
Magnitude	20%	0.15	0.02	9.76	<0.01	0.12	0.19
Consequence	Moderate	0.00					
Consequence	Severe	0.04	0.01	3.19	<0.01	0.01	0.06
Availability	High	0.00					
Availability	Low	-0.01	0.01	-0.44	0.66	-0.03	0.02
Default	None	0.00					
Default	ETA	-0.02	0.01	-1.50	0.13	-0.05	0.01
Default	EIA	0.01	0.01	0.78	0.43	-0.02	0.04

Table S8C. AMCE – Republicans

Attribute	Level	Estimate	SE	z	p	Lower CI	Upper CI
Scenario	Healthcare	0.00					
Scenario	Lending	0.03	0.02	1.78	0.08	0.00	0.06
Scenario	Insurance	0.01	0.02	0.91	0.36	-0.02	0.04
ETA Accuracy	70%	0.00					
ETA Accuracy	80%	0.01	0.02	0.30	0.76	-0.03	0.04
ETA Accuracy	90%	0.01	0.02	0.29	0.77	-0.03	0.04
Accuracy Loss	5%	0.00					
Accuracy Loss	10%	-0.07	0.02	-3.45	<0.01	-0.11	-0.03
Accuracy Loss	20%	-0.21	0.02	-9.93	<0.01	-0.26	-0.17
Disadvantaged Group	Women	0.00					
Disadvantaged Group	POC	0.01	0.02	0.59	0.56	-0.02	0.05
Disadvantaged Group	PWD	-0.02	0.02	-1.08	0.28	-0.05	0.02
Magnitude	5%	0.00					
Magnitude	10%	0.04	0.02	2.05	0.04	0.00	0.07
Magnitude	20%	0.10	0.02	5.00	<0.01	0.06	0.14
Consequence	Moderate	0.00					
Consequence	Severe	0.04	0.01	2.83	<0.01	0.01	0.07
Availability	High	0.00					
Availability	Low	0.02	0.01	1.26	0.21	-0.01	0.05
Default	None	0.00					
Default	ETA	0.00	0.02	0.21	0.83	-0.03	0.04
Default	EIA	0.01	0.02	0.82	0.41	-0.02	0.05

Table S9A. Difference in AMCE – Independents - Democrats

**Attribute**	**Level**	**Estimate**	**SE**	***z***	***p***	**Lower CI**	**Upper CI**
Scenario	Lending	0.01	0.02	0.84	0.40	-0.02	0.05
Scenario	Insurance	0.01	0.02	0.71	0.48	-0.02	0.04
ETA Accuracy	80%	0.04	0.02	2.46	0.01	0.01	0.08
ETA Accuracy	90%	0.01	0.02	0.59	0.56	-0.03	0.05
Accuracy Loss	10%	-0.01	0.02	-0.48	0.63	-0.05	0.03
Accuracy Loss	20%	-0.02	0.02	-1.13	0.26	-0.07	0.02
Disadvantaged Group	POC	0.00	0.02	0.17	0.87	-0.03	0.04
Disadvantaged Group	PWD	-0.01	0.02	-0.45	0.65	-0.04	0.03
Magnitude	10%	-0.03	0.02	-1.58	0.11	-0.07	0.01
Magnitude	20%	0.00	0.02	0.13	0.90	-0.04	0.04
Consequence	Severe	0.02	0.02	1.03	0.30	-0.01	0.05
Availability	Low	0.00	0.02	-0.06	0.95	-0.03	0.03
Default	ETA	-0.01	0.02	-0.45	0.65	-0.04	0.03
Default	EIA	0.00	0.02	-0.23	0.82	-0.04	0.03

Table S9B. Difference in AMCE – Republicans - Democrats

**Attribute**	**Level**	**Estimate**	**SE**	***z***	***p***	**Lower CI**	**Upper CI**
Scenario	Lending	0.00	0.02	-0.17	0.87	-0.04	0.03
Scenario	Insurance	-0.01	0.02	-0.37	0.71	-0.04	0.03
ETA Accuracy	80%	-0.01	0.02	-0.62	0.54	-0.05	0.03
ETA Accuracy	90%	-0.02	0.02	-0.97	0.33	-0.06	0.02
Accuracy Loss	10%	0.01	0.02	0.63	0.53	-0.03	0.06
Accuracy Loss	20%	0.04	0.03	1.59	0.11	-0.01	0.09
Disadvantaged Group	POC	-0.03	0.02	-1.55	0.12	-0.07	0.01
Disadvantaged Group	PWD	-0.02	0.02	-0.86	0.39	-0.06	0.02
Magnitude	10%	-0.04	0.02	-1.89	0.06	-0.08	0.00
Magnitude	20%	-0.05	0.02	-2.32	0.02	-0.10	-0.01
Consequence	Severe	0.02	0.02	1.07	0.28	-0.02	0.05
Availability	Low	0.02	0.02	1.35	0.18	-0.01	0.06
Default	ETA	0.02	0.02	0.79	0.43	-0.02	0.06
Default	EIA	-0.00	0.02	-0.04	0.96	-0.04	0.04

Figure S1. Subgroup Analysis – Equality Orientation

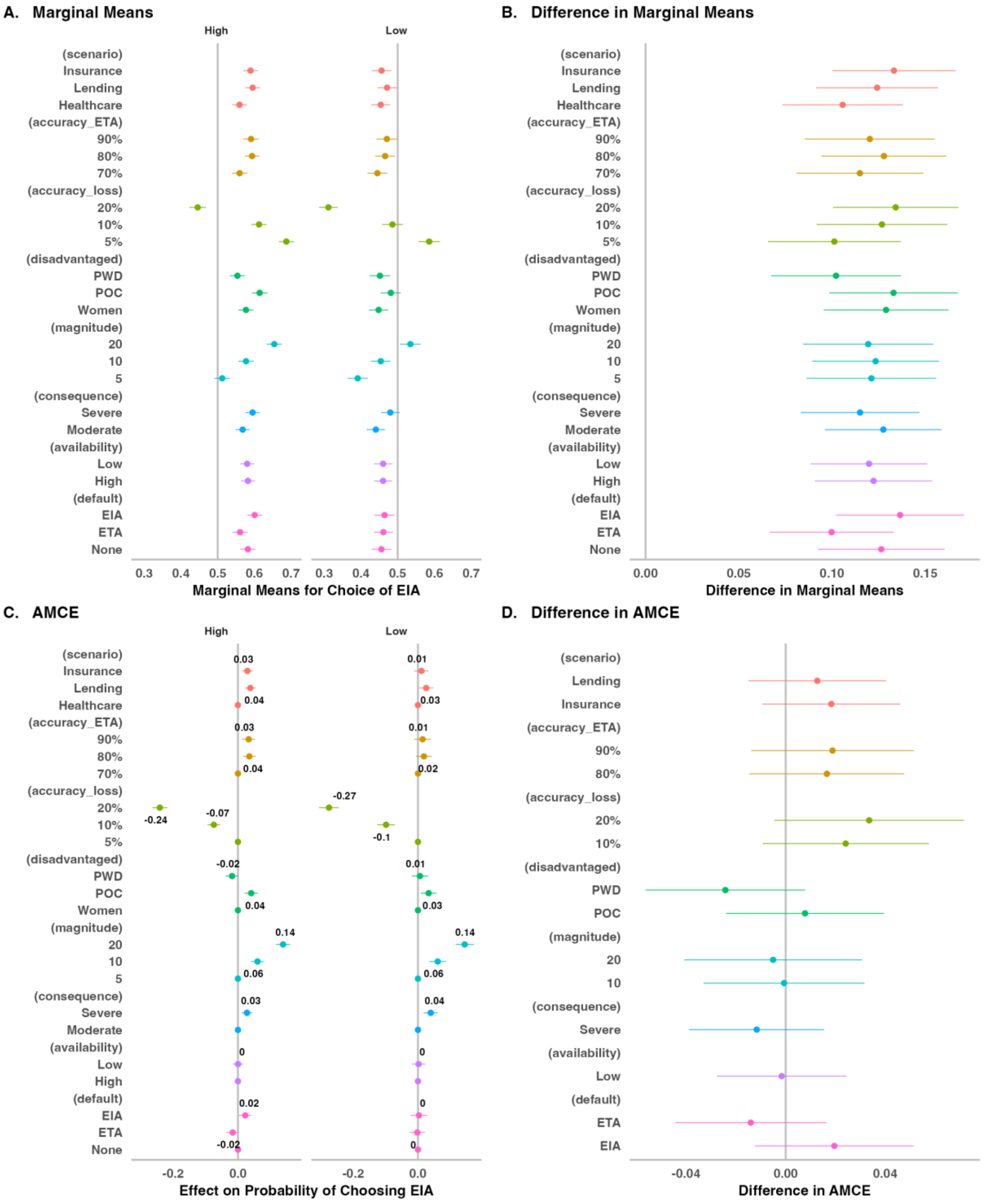

Note. Scores of 3 or lower on the equality dimension of the BJSO scale were coded as low equality orientation. Scores higher than 3 were coded as high equality orientation.

Table S10A. Marginal Means – Low Equality Orientation

Attribute	Level	Estimate	SE	Lower CI	Upper CI
Scenario	Healthcare	0.45	0.01	0.43	0.48
Scenario	Lending	0.47	0.01	0.45	0.50
Scenario	Insurance	0.46	0.01	0.43	0.48
ETA Accuracy	70%	0.44	0.01	0.42	0.47
ETA Accuracy	80%	0.47	0.01	0.44	0.49
ETA Accuracy	90%	0.47	0.01	0.44	0.50
Accuracy Loss	5%	0.59	0.01	0.56	0.61
Accuracy Loss	10%	0.49	0.01	0.46	0.51
Accuracy Loss	20%	0.31	0.01	0.29	0.34
Disadvantaged Group	Women	0.45	0.01	0.42	0.47
Disadvantaged Group	POC	0.48	0.01	0.45	0.51
Disadvantaged Group	PWD	0.45	0.01	0.42	0.48
Magnitude	5%	0.39	0.01	0.36	0.42
Magnitude	10%	0.45	0.01	0.43	0.48
Magnitude	20%	0.53	0.01	0.51	0.56
Consequence	Moderate	0.44	0.01	0.42	0.47
Consequence	Severe	0.48	0.01	0.45	0.51
Availability	High	0.46	0.01	0.44	0.49
Availability	Low	0.46	0.01	0.44	0.49
Default	None	0.46	0.01	0.43	0.48
Default	ETA	0.46	0.01	0.43	0.49
Default	EIA	0.46	0.01	0.44	0.49

Table S10B. Marginal Means – High Equality Orientation

Attribute	Level	Estimate	SE	Lower CI	Upper CI
Scenario	Healthcare	0.56	0.01	0.54	0.58
Scenario	Lending	0.60	0.01	0.58	0.62
Scenario	Insurance	0.59	0.01	0.57	0.61
ETA Accuracy	70%	0.56	0.01	0.54	0.58
ETA Accuracy	80%	0.59	0.01	0.57	0.61
ETA Accuracy	90%	0.59	0.01	0.57	0.61
Accuracy Loss	5%	0.69	0.01	0.67	0.71
Accuracy Loss	10%	0.61	0.01	0.59	0.63
Accuracy Loss	20%	0.45	0.01	0.42	0.47
Disadvantaged Group	Women	0.58	0.01	0.56	0.60
Disadvantaged Group	POC	0.61	0.01	0.59	0.64
Disadvantaged Group	PWD	0.55	0.01	0.53	0.57
Magnitude	5%	0.51	0.01	0.49	0.53
Magnitude	10%	0.58	0.01	0.56	0.60
Magnitude	20%	0.65	0.01	0.63	0.67
Consequence	Moderate	0.57	0.01	0.55	0.59
Consequence	Severe	0.60	0.01	0.58	0.61
Availability	High	0.58	0.01	0.56	0.60
Availability	Low	0.58	0.01	0.56	0.60
Default	None	0.58	0.01	0.56	0.60
Default	ETA	0.56	0.01	0.54	0.58
Default	EIA	0.60	0.01	0.58	0.62

Table S11. Difference in Marginal Means – High - Low Equality Orientation

**Attribute**	**Level**	**Estimate**	**SE**	***z***	***p***	**Lower CI**	**Upper CI**
Scenario	Healthcare	0.11	0.02	6.41	<0.01	0.07	0.14
Scenario	Lending	0.12	0.02	7.44	<0.01	0.09	0.16
Scenario	Insurance	0.13	0.02	7.90	<0.01	0.10	0.17
ETA Accuracy	70%	0.11	0.02	6.63	<0.01	0.08	0.15
ETA Accuracy	80%	0.13	0.02	7.49	<0.01	0.09	0.16
ETA Accuracy	90%	0.12	0.02	6.76	<0.01	0.09	0.16
Accuracy Loss	5%	0.10	0.02	5.58	<0.01	0.07	0.14
Accuracy Loss	10%	0.13	0.02	7.09	<0.01	0.09	0.16
Accuracy Loss	20%	0.13	0.02	7.81	<0.01	0.10	0.17
Disadvantaged Group	Women	0.13	0.02	7.55	<0.01	0.10	0.16
Disadvantaged Group	POC	0.13	0.02	7.55	<0.01	0.10	0.17
Disadvantaged Group	PWD	0.10	0.02	5.75	<0.01	0.07	0.14
Magnitude	5%	0.12	0.02	6.83	<0.01	0.09	0.16
Magnitude	10%	0.12	0.02	7.10	<0.01	0.09	0.16
Magnitude	20%	0.12	0.02	6.70	<0.01	0.08	0.15
Consequence	Moderate	0.13	0.02	7.98	<0.01	0.10	0.16
Consequence	Severe	0.12	0.02	7.08	<0.01	0.08	0.15
Availability	High	0.12	0.02	7.61	<0.01	0.09	0.15
Availability	Low	0.12	0.02	7.52	<0.01	0.09	0.15
Default	None	0.13	0.02	7.30	<0.01	0.09	0.16
Default	ETA	0.10	0.02	5.88	<0.01	0.07	0.13
Default	EIA	0.14	0.02	7.82	<0.01	0.10	0.17

Table S12A. AMCE – Low Equality Orientation

Attribute	Level	Estimate	SE	*z*	*p*	Lower CI	Upper CI
Scenario	Healthcare	0.00					
Scenario	Lending	0.03	0.01	2.32	0.02	0.00	0.05
Scenario	Insurance	0.01	0.01	0.96	0.34	-0.01	0.03
ETA Accuracy	70%	0.00					
ETA Accuracy	80%	0.02	0.01	1.49	0.14	-0.01	0.04
ETA Accuracy	90%	0.01	0.01	1.09	0.27	-0.01	0.04
Accuracy Loss	5%	0.00					
Accuracy Loss	10%	-0.10	0.01	-7.11	<0.01	-0.13	-0.07
Accuracy Loss	20%	-0.27	0.02	-17.72	<0.01	-0.31	-0.24
Disadvantaged Group	Women	0.00					
Disadvantaged Group	POC	0.03	0.01	2.63	0.01	0.01	0.06
Disadvantaged Group	PWD	0.01	0.01	0.47	0.64	-0.02	0.03
Magnitude	5%	0.00					
Magnitude	10%	0.06	0.01	4.72	<0.01	0.04	0.09
Magnitude	20%	0.14	0.01	10.02	<0.01	0.12	0.17
Consequence	Moderate	0.00			<0.01		
Consequence	Severe	0.04	0.01	3.54	<0.01	0.02	0.06
Availability	High	0.00					
Availability	Low	0.00	0.01	0.18	0.85	-0.02	0.02
Default	None	0.00					
Default	ETA	0.00	0.01	-0.17	0.87	-0.03	0.02
Default	EIA	0.00	0.01	0.23	0.82	-0.02	0.03

Table S12B. AMCE – High Equality Orientation

**Attribute**	**Level**	**Estimate**	**SE**	***z***	***p***	**Lower CI**	**Upper CI**
Scenario	Healthcare	0.00					
Scenario	Lending	0.04	0.01	4.29	<0.01	0.02	0.06
Scenario	Insurance	0.03	0.01	3.42	<0.01	0.01	0.05
ETA Accuracy	70%	0.00			<0.01		
ETA Accuracy	80%	0.04	0.01	3.57	<0.01	0.02	0.05
ETA Accuracy	90%	0.03	0.01	3.23	<0.01	0.01	0.05
Accuracy Loss	5%	0.00			<0.01		
Accuracy Loss	10%	-0.07	0.01	-7.55	<0.01	-0.09	-0.06
Accuracy Loss	20%	-0.24	0.01	-20.67	<0.01	-0.26	-0.22
Disadvantaged Group	Women	0.00			<0.01		
Disadvantaged Group	POC	0.04	0.01	4.00	<0.01	0.02	0.06
Disadvantaged Group	PWD	-0.02	0.01	-1.79	0.07	-0.04	0.00
Magnitude	5%	0.00					
Magnitude	10%	0.06	0.01	5.90	<0.01	0.04	0.08
Magnitude	20%	0.14	0.01	12.49	<0.01	0.12	0.16
Consequence	Moderate	0.00			<0.01		
Consequence	Severe	0.03	0.01	3.40	<0.01	0.01	0.04
Availability	High	0.00					
Availability	Low	0.00	0.01	0.04	0.97	-0.02	0.02
Default	None	0.00					
Default	ETA	-0.02	0.01	-1.65	0.10	-0.04	0.00
Default	EIA	0.02	0.01	2.33	0.02	0.00	0.04

Table S13. Difference in AMCE – High - Low Equality Orientation

Attribute	Level	Estimate	SE	*z*	*p*	Lower CI	Upper CI
Scenario	Lending	0.01	0.01	0.90	0.37	-0.01	0.04
Scenario	Insurance	0.02	0.01	1.29	0.20	-0.01	0.05
ETA Accuracy	80%	0.02	0.02	1.04	0.30	-0.01	0.05
ETA Accuracy	90%	0.02	0.02	1.13	0.26	-0.01	0.05
Accuracy Loss	10%	0.02	0.02	1.42	0.16	-0.01	0.06
Accuracy Loss	20%	0.03	0.02	1.73	0.08	0.00	0.07
Disadvantaged Group	POC	0.01	0.02	0.48	0.63	-0.02	0.04
Disadvantaged Group	PWD	-0.02	0.02	-1.48	0.14	-0.06	0.01
Magnitude	10%	0.00	0.02	-0.04	0.97	-0.03	0.03
Magnitude	20%	-0.01	0.02	-0.28	0.78	-0.04	0.03
Consequence	Severe	-0.01	0.01	-0.84	0.40	-0.04	0.02
Availability	Low	0.00	0.01	-0.12	0.90	-0.03	0.02
Default	ETA	-0.01	0.02	-0.91	0.36	-0.04	0.02
Default	EIA	0.02	0.02	1.20	0.23	-0.01	0.05

Figure S2. Subgroup Analysis Equity Orientation

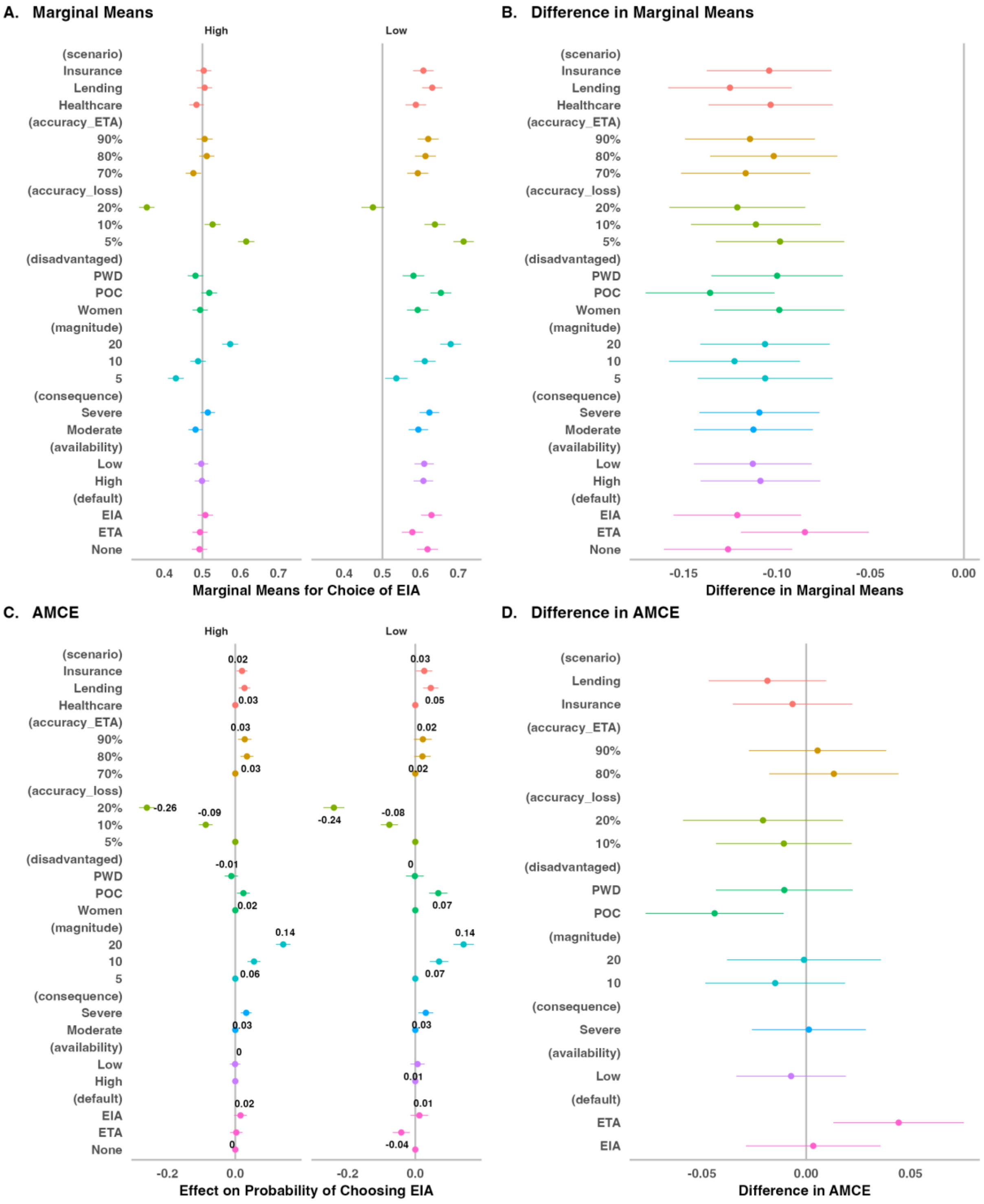

Note. Scores of 3 or lower on the equity dimension of the BJSO scale were coded as low equity orientation. Scores higher than 3 were coded as high equity orientation.

Table S14A. Marginal Means – Low Equity Orientation

Attribute	Level	Estimate	SE	Lower CI	Upper CI
Scenario	Healthcare	0.59	0.01	0.56	0.61
Scenario	Lending	0.63	0.01	0.60	0.66
Scenario	Insurance	0.61	0.01	0.58	0.63
ETA Accuracy	70%	0.59	0.01	0.57	0.62
ETA Accuracy	80%	0.61	0.01	0.59	0.64
ETA Accuracy	90%	0.62	0.01	0.59	0.65
Accuracy Loss	5%	0.71	0.01	0.69	0.74
Accuracy Loss	10%	0.64	0.01	0.61	0.67
Accuracy Loss	20%	0.47	0.02	0.44	0.51
Disadvantaged Group	Women	0.59	0.01	0.56	0.62
Disadvantaged Group	POC	0.65	0.01	0.63	0.68
Disadvantaged Group	PWD	0.58	0.01	0.55	0.61
Magnitude	5%	0.54	0.02	0.51	0.57
Magnitude	10%	0.61	0.01	0.58	0.64
Magnitude	20%	0.68	0.01	0.65	0.71
Consequence	Moderate	0.59	0.01	0.57	0.62
Consequence	Severe	0.62	0.01	0.60	0.65
Availability	High	0.61	0.01	0.58	0.63
Availability	Low	0.61	0.01	0.58	0.64
Default	None	0.62	0.01	0.59	0.65
Default	ETA	0.58	0.01	0.55	0.61
Default	EIA	0.63	0.01	0.60	0.66

Table S14B. Marginal Means – High Equity Orientation

Attribute	Level	Estimate	SE	Lower CI	Upper CI
Scenario	Healthcare	0.48	0.01	0.47	0.50
Scenario	Lending	0.51	0.01	0.49	0.53
Scenario	Insurance	0.50	0.01	0.48	0.52
ETA Accuracy	70%	0.48	0.01	0.46	0.50
ETA Accuracy	80%	0.51	0.01	0.49	0.53
ETA Accuracy	90%	0.51	0.01	0.48	0.53
Accuracy Loss	5%	0.62	0.01	0.59	0.64
Accuracy Loss	10%	0.53	0.01	0.51	0.55
Accuracy Loss	20%	0.35	0.01	0.33	0.37
Disadvantaged Group	Women	0.49	0.01	0.47	0.51
Disadvantaged Group	POC	0.52	0.01	0.50	0.54
Disadvantaged Group	PWD	0.48	0.01	0.46	0.50
Magnitude	5%	0.43	0.01	0.41	0.45
Magnitude	10%	0.49	0.01	0.47	0.51
Magnitude	20%	0.57	0.01	0.55	0.59
Consequence	Moderate	0.48	0.01	0.46	0.50
Consequence	Severe	0.51	0.01	0.49	0.53
Availability	High	0.50	0.01	0.48	0.52
Availability	Low	0.50	0.01	0.48	0.52
Default	None	0.49	0.01	0.47	0.51
Default	ETA	0.49	0.01	0.47	0.51
Default	EIA	0.51	0.01	0.49	0.53

Table S15. Difference in Marginal Means – High - Low Equity Orientation

Attribute	Level	Estimate	SE	*z*	*p*	Lower CI	Upper CI
Scenario	Healthcare	-0.10	0.02	-6.10	<0.01	-0.14	-0.07
Scenario	Lending	-0.13	0.02	-7.42	<0.01	-0.16	-0.09
Scenario	Insurance	-0.10	0.02	-6.11	<0.01	-0.14	-0.07
ETA Accuracy	70%	-0.12	0.02	-6.62	<0.01	-0.15	-0.08
ETA Accuracy	80%	-0.10	0.02	-5.86	<0.01	-0.14	-0.07
ETA Accuracy	90%	-0.11	0.02	-6.46	<0.01	-0.15	-0.08
Accuracy Loss	5%	-0.10	0.02	-5.61	<0.01	-0.13	-0.06
Accuracy Loss	10%	-0.11	0.02	-6.28	<0.01	-0.15	-0.08
Accuracy Loss	20%	-0.12	0.02	-6.54	<0.01	-0.16	-0.08
Disadvantaged Group	Women	-0.10	0.02	-5.57	<0.01	-0.13	-0.06
Disadvantaged Group	POC	-0.14	0.02	-7.71	<0.01	-0.17	-0.10
Disadvantaged Group	PWD	-0.10	0.02	-5.57	<0.01	-0.14	-0.06
Magnitude	5%	-0.11	0.02	-5.77	<0.01	-0.14	-0.07
Magnitude	10%	-0.12	0.02	-6.87	<0.01	-0.16	-0.09
Magnitude	20%	-0.11	0.02	-6.02	<0.01	-0.14	-0.07
Consequence	Moderate	-0.11	0.02	-6.94	<0.01	-0.14	-0.08
Consequence	Severe	-0.11	0.02	-6.67	<0.01	-0.14	-0.08
Availability	High	-0.11	0.02	-6.66	<0.01	-0.14	-0.08
Availability	Low	-0.11	0.02	-7.02	<0.01	-0.14	-0.08
Default	None	-0.13	0.02	-7.21	<0.01	-0.16	-0.09
Default	ETA	-0.09	0.02	-4.87	<0.01	-0.12	-0.05
Default	EIA	-0.12	0.02	-6.97	<0.01	-0.16	-0.09

Table S16A. AMCE – Low Equity Orientation

**Attribute**	**Level**	**Estimate**	**SE**	***z***	***p***	**Lower CI**	**Upper CI**
Scenario	Healthcare	0.00					
Scenario	Lending	0.05	0.01	3.97	<0.01	0.02	0.07
Scenario	Insurance	0.03	0.01	2.19	0.03	0.00	0.05
ETA Accuracy	70%	0.00					
ETA Accuracy	80%	0.02	0.01	1.69	0.09	0.00	0.05
ETA Accuracy	90%	0.02	0.01	1.66	0.10	0.00	0.05
Accuracy Loss	5%	0.00					
Accuracy Loss	10%	-0.08	0.01	-5.91	<0.01	-0.10	-0.05
Accuracy Loss	20%	-0.24	0.02	-15.26	<0.01	-0.27	-0.21
Disadvantaged Group	Women	0.00			<0.01		
Disadvantaged Group	POC	0.07	0.01	4.89	<0.01	0.04	0.10
Disadvantaged Group	PWD	0.00	0.01	-0.09	0.93	-0.03	0.03
Magnitude	5%	0.00					
Magnitude	10%	0.07	0.01	5.01	<0.01	0.04	0.10
Magnitude	20%	0.14	0.02	9.25	<0.01	0.11	0.17
Consequence	Moderate	0.00					
Consequence	Severe	0.03	0.01	2.74	0.01	0.01	0.05
Availability	High	0.00					
Availability	Low	0.01	0.01	0.62	0.53	-0.01	0.03
Default	None	0.00					
Default	ETA	-0.04	0.01	-3.20	<0.01	-0.07	-0.02
Default	EIA	0.01	0.01	0.89	0.37	-0.01	0.04

Table S16B. AMCE – High Equity Orientation

Attribute	Level	Estimate	SE	*z*	*p*	Lower CI	Upper CI
Scenario	Healthcare	0.00					
Scenario	Lending	0.03	0.01	3.15	<0.01	0.01	0.04
Scenario	Insurance	0.02	0.01	2.41	0.02	0.00	0.04
ETA Accuracy	70%	0.00					
ETA Accuracy	80%	0.03	0.01	3.51	<0.01	0.02	0.05
ETA Accuracy	90%	0.03	0.01	2.75	0.01	0.01	0.05
Accuracy Loss	5%	0.00					
Accuracy Loss	10%	-0.09	0.01	-8.39	<0.01	-0.11	-0.07
Accuracy Loss	20%	-0.26	0.01	-22.41	<0.01	-0.28	-0.24
Disadvantaged Group	Women	0.00					
Disadvantaged Group	POC	0.02	0.01	2.47	0.01	0.00	0.04
Disadvantaged Group	PWD	-0.01	0.01	-1.18	0.24	-0.03	0.01
Magnitude	5%	0.00					
Magnitude	10%	0.06	0.01	5.62	<0.01	0.04	0.07
Magnitude	20%	0.14	0.01	13.03	<0.01	0.12	0.16
Consequence	Moderate	0.00					
Consequence	Severe	0.03	0.01	3.93	<0.01	0.02	0.05
Availability	High	0.00					
Availability	Low	0.00	0.01	-0.07	0.94	-0.02	0.02
Default	None	0.00					
Default	ETA	0.00	0.01	0.31	0.76	-0.02	0.02
Default	EIA	0.02	0.01	1.61	0.11	0.00	0.03

Table S17. Difference in AMCE – High - Low Equity Orientation

**Attribute**	**Level**	**Estimate**	**SE**	***z***	***p***	**Lower CI**	**Upper CI**
Scenario	Lending	-0.02	0.01	-1.29	0.20	-0.05	0.01
Scenario	Insurance	-0.01	0.01	-0.45	0.65	-0.04	0.02
ETA Accuracy	80%	0.01	0.02	0.84	0.40	-0.02	0.04
ETA Accuracy	90%	0.01	0.02	0.32	0.75	-0.03	0.04
Accuracy Loss	10%	-0.01	0.02	-0.65	0.52	-0.04	0.02
Accuracy Loss	20%	-0.02	0.02	-1.06	0.29	-0.06	0.02
Disadvantaged Group	POC	-0.04	0.02	-2.60	0.01	-0.08	-0.01
Disadvantaged Group	PWD	-0.01	0.02	-0.63	0.53	-0.04	0.02
Magnitude	10%	-0.01	0.02	-0.87	0.38	-0.05	0.02
Magnitude	20%	0.00	0.02	-0.06	0.95	-0.04	0.04
Consequence	Severe	0.00	0.01	0.09	0.93	-0.03	0.03
Availability	Low	-0.01	0.01	-0.54	0.59	-0.03	0.02
Default	ETA	0.04	0.02	2.78	0.01	0.01	0.08
Default	EIA	0.00	0.02	0.21	0.84	-0.03	0.04

Figure S3. Subgroup Analysis Need Orientation

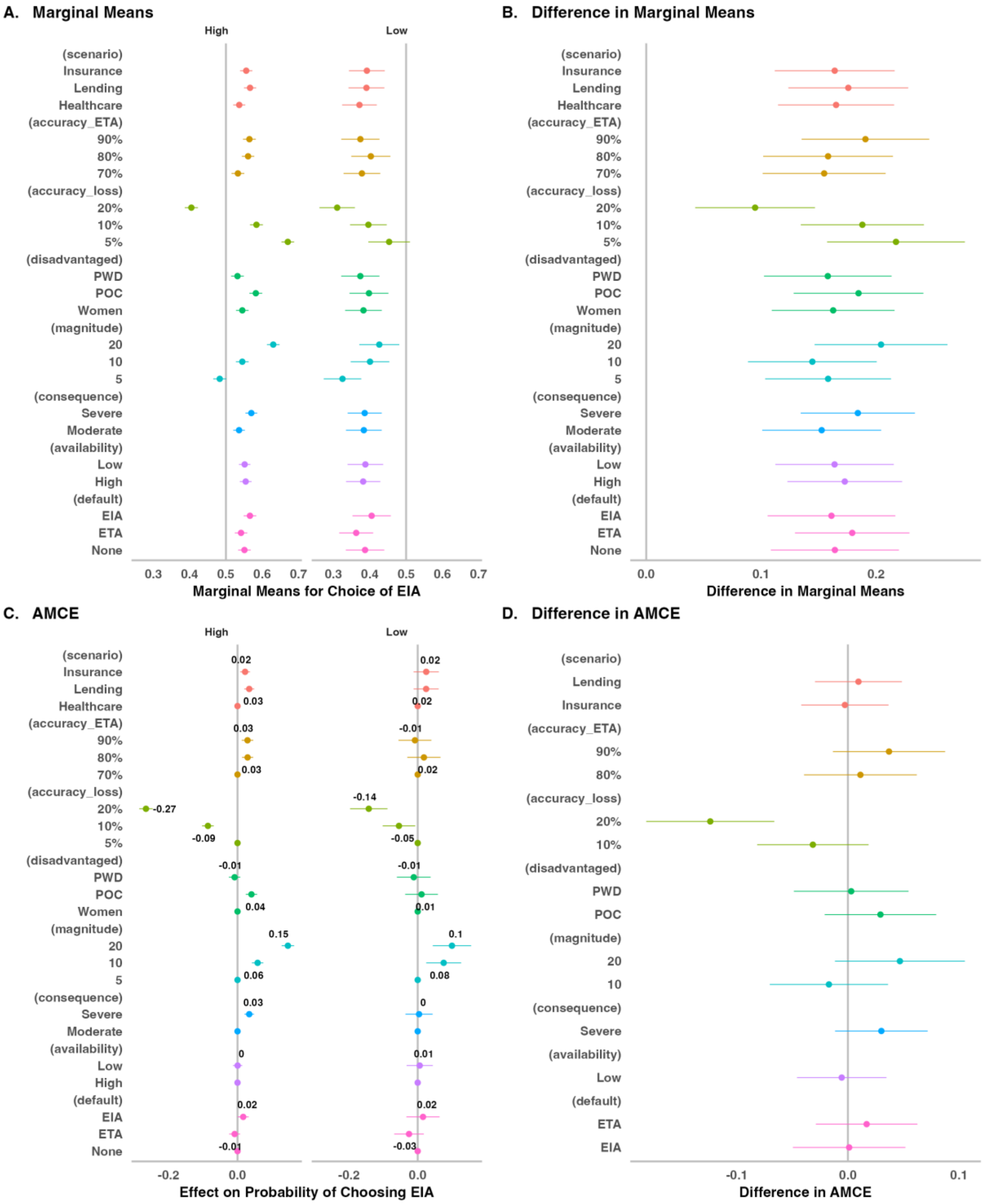

Note. Scores of 3 or lower on the need dimension of the BJSO scale were coded as low need orientation. Scores higher than 3 were coded as high need orientation.

Table S18A. Marginal Means – Low Need Orientation

Attribute	Level	Estimate	SE	Lower CI	Upper CI
Scenario	Healthcare	0.37	0.02	0.32	0.42
Scenario	Lending	0.39	0.03	0.34	0.44
Scenario	Insurance	0.39	0.03	0.34	0.44
ETA Accuracy	70%	0.38	0.03	0.33	0.43
ETA Accuracy	80%	0.40	0.03	0.35	0.46
ETA Accuracy	90%	0.37	0.03	0.32	0.43
Accuracy Loss	5%	0.45	0.03	0.40	0.51
Accuracy Loss	10%	0.40	0.03	0.35	0.45
Accuracy Loss	20%	0.31	0.02	0.26	0.36
Disadvantaged Group	Women	0.38	0.03	0.33	0.43
Disadvantaged Group	POC	0.40	0.03	0.34	0.45
Disadvantaged Group	PWD	0.37	0.03	0.32	0.43
Magnitude	5%	0.32	0.03	0.27	0.38
Magnitude	10%	0.40	0.03	0.35	0.45
Magnitude	20%	0.43	0.03	0.37	0.48
Consequence	Moderate	0.38	0.03	0.33	0.43
Consequence	Severe	0.39	0.02	0.34	0.43
Availability	High	0.38	0.02	0.33	0.43
Availability	Low	0.39	0.03	0.34	0.44
Default	None	0.39	0.03	0.33	0.44
Default	ETA	0.36	0.02	0.32	0.41
Default	EIA	0.40	0.03	0.35	0.46

Table S18B. Marginal Means – High Need Orientation

Attribute	Level	Estimate	SE	Lower CI	Upper CI
Scenario	Healthcare	0.54	0.01	0.52	0.55
Scenario	Lending	0.57	0.01	0.55	0.58
Scenario	Insurance	0.56	0.01	0.54	0.57
ETA Accuracy	70%	0.53	0.01	0.52	0.55
ETA Accuracy	80%	0.56	0.01	0.54	0.58
ETA Accuracy	90%	0.56	0.01	0.55	0.58
Accuracy Loss	5%	0.67	0.01	0.65	0.69
Accuracy Loss	10%	0.58	0.01	0.57	0.60
Accuracy Loss	20%	0.40	0.01	0.39	0.42
Disadvantaged Group	Women	0.55	0.01	0.53	0.56
Disadvantaged Group	POC	0.58	0.01	0.56	0.60
Disadvantaged Group	PWD	0.53	0.01	0.51	0.55
Magnitude	5%	0.48	0.01	0.46	0.50
Magnitude	10%	0.55	0.01	0.53	0.56
Magnitude	20%	0.63	0.01	0.61	0.65
Consequence	Moderate	0.54	0.01	0.52	0.55
Consequence	Severe	0.57	0.01	0.55	0.59
Availability	High	0.55	0.01	0.54	0.57
Availability	Low	0.55	0.01	0.54	0.57
Default	None	0.55	0.01	0.53	0.57
Default	ETA	0.54	0.01	0.52	0.56
Default	EIA	0.57	0.01	0.55	0.58

Table S19. Difference in Marginal Means – High - Low Need Orientation

Attribute	Level	Estimate	SE	*z*	*p*	Lower CI	Upper CI
Scenario	Healthcare	0.17	0.03	6.41	<0.01	0.11	0.22
Scenario	Lending	0.18	0.03	6.61	<0.01	0.12	0.23
Scenario	Insurance	0.16	0.03	6.16	<0.01	0.11	0.22
ETA Accuracy	70%	0.15	0.03	5.67	<0.01	0.10	0.21
ETA Accuracy	80%	0.16	0.03	5.49	<0.01	0.10	0.21
ETA Accuracy	90%	0.19	0.03	6.73	<0.01	0.14	0.25
Accuracy Loss	5%	0.22	0.03	7.11	<0.01	0.16	0.28
Accuracy Loss	10%	0.19	0.03	6.88	<0.01	0.13	0.24
Accuracy Loss	20%	0.09	0.03	3.56	<0.01	0.04	0.15
Disadvantaged Group	Women	0.16	0.03	5.97	<0.01	0.11	0.22
Disadvantaged Group	POC	0.18	0.03	6.42	<0.01	0.13	0.24
Disadvantaged Group	PWD	0.16	0.03	5.58	<0.01	0.10	0.21
Magnitude	5%	0.16	0.03	5.67	<0.01	0.10	0.21
Magnitude	10%	0.14	0.03	5.06	<0.01	0.09	0.20
Magnitude	20%	0.20	0.03	6.93	<0.01	0.15	0.26
Consequence	Moderate	0.15	0.03	5.78	<0.01	0.10	0.20
Consequence	Severe	0.18	0.03	7.26	<0.01	0.13	0.23
Availability	High	0.17	0.03	6.79	<0.01	0.12	0.22
Availability	Low	0.16	0.03	6.23	<0.01	0.11	0.22
Default	None	0.16	0.03	5.77	<0.01	0.11	0.22
Default	ETA	0.18	0.03	7.06	<0.01	0.13	0.23
Default	EIA	0.16	0.03	5.69	<0.01	0.11	0.22

Table S20A. AMCE – Low Need Orientation

Attribute	Level	Estimate	SE	*z*	*p*	Lower CI	Upper CI
Scenario	Healthcare	0.00					
Scenario	Lending	0.02	0.02	1.32	0.19	-0.01	0.06
Scenario	Insurance	0.02	0.02	1.32	0.19	-0.01	0.06
ETA Accuracy	70%	0.00					
ETA Accuracy	80%	0.02	0.02	0.73	0.46	-0.03	0.07
ETA Accuracy	90%	-0.01	0.02	-0.33	0.74	-0.06	0.04
Accuracy Loss	5%	0.00					
Accuracy Loss	10%	-0.05	0.02	-2.26	0.02	-0.10	-0.01
Accuracy Loss	20%	-0.14	0.03	-5.13	<0.01	-0.20	-0.09
Disadvantaged Group	Women	0.00					
Disadvantaged Group	POC	0.01	0.02	0.46	0.64	-0.04	0.06
Disadvantaged Group	PWD	-0.01	0.03	-0.46	0.65	-0.06	0.04
Magnitude	5%	0.00					
Magnitude	10%	0.08	0.03	2.92	<0.01	0.02	0.13
Magnitude	20%	0.10	0.03	3.50	<0.01	0.04	0.16
Consequence	Moderate	0.00					
Consequence	Severe	0.00	0.02	0.19	0.85	-0.04	0.04
Availability	High	0.00					
Availability	Low	0.01	0.02	0.30	0.76	-0.03	0.04
Default	None	0.00					
Default	ETA	-0.03	0.02	-1.15	0.25	-0.07	0.02
Default	EIA	0.02	0.02	0.63	0.53	-0.03	0.06

Table S20B. AMCE – High Need Orientation

Attribute	Level	Estimate	SE	*z*	*p*	Lower CI	Upper CI
Scenario	Healthcare	0.00					
Scenario	Lending	0.03	0.01	4.59	<0.01	0.02	0.05
Scenario	Insurance	0.02	0.01	3.02	<0.01	0.01	0.04
ETA Accuracy	70%	0.00			<0.01		
ETA Accuracy	80%	0.03	0.01	3.56	<0.01	0.01	0.05
ETA Accuracy	90%	0.03	0.01	3.39	<0.01	0.01	0.05
Accuracy Loss	5%	0.00			<0.01		
Accuracy Loss	10%	-0.09	0.01	-9.96	<0.01	-0.10	-0.07
Accuracy Loss	20%	-0.27	0.01	-26.79	<0.01	-0.29	-0.25
Disadvantaged Group	Women	0.00			<0.01		
Disadvantaged Group	POC	0.04	0.01	4.82	<0.01	0.02	0.06
Disadvantaged Group	PWD	-0.01	0.01	-1.01	0.31	-0.03	0.01
Magnitude	5%	0.00					
Magnitude	10%	0.06	0.01	6.91	<0.01	0.04	0.07
Magnitude	20%	0.15	0.01	15.82	<0.01	0.13	0.16
Consequence	Moderate	0.00			<0.01		
Consequence	Severe	0.03	0.01	4.86	<0.01	0.02	0.05
Availability	High	0.00					
Availability	Low	0.00	0.01	0.05	0.96	-0.01	0.01
Default	None	0.00					
Default	ETA	-0.01	0.01	-1.04	0.30	-0.02	0.01
Default	EIA	0.02	0.01	2.02	0.04	0.00	0.03

Table S21. Difference in AMCE – High - Low Need Orientation

**Attribute**	**Level**	**Estimate**	**SE**	***z***	***p***	**Lower CI**	**Upper CI**
Scenario	Lending	0.01	0.02	0.48	0.63	-0.03	0.05
Scenario	Insurance	0.00	0.02	-0.14	0.89	-0.04	0.04
ETA Accuracy	80%	0.01	0.03	0.43	0.66	-0.04	0.06
ETA Accuracy	90%	0.04	0.03	1.44	0.15	-0.01	0.09
Accuracy Loss	10%	-0.03	0.03	-1.23	0.22	-0.08	0.02
Accuracy Loss	20%	-0.12	0.03	-4.21	0.00	-0.18	-0.07
Disadvantaged Group	POC	0.03	0.03	1.14	0.25	-0.02	0.08
Disadvantaged Group	PWD	0.00	0.03	0.11	0.91	-0.05	0.05
Magnitude	10%	-0.02	0.03	-0.63	0.53	-0.07	0.04
Magnitude	20%	0.05	0.03	1.57	0.12	-0.01	0.11
Consequence	Severe	0.03	0.02	1.42	0.16	-0.01	0.07
Availability	Low	-0.01	0.02	-0.27	0.79	-0.05	0.03
Default	ETA	0.02	0.02	0.72	0.47	-0.03	0.06
Default	EIA	0.00	0.03	0.04	0.97	-0.05	0.05

Figure S4. Subgroup Analysis Entitlement Orientation

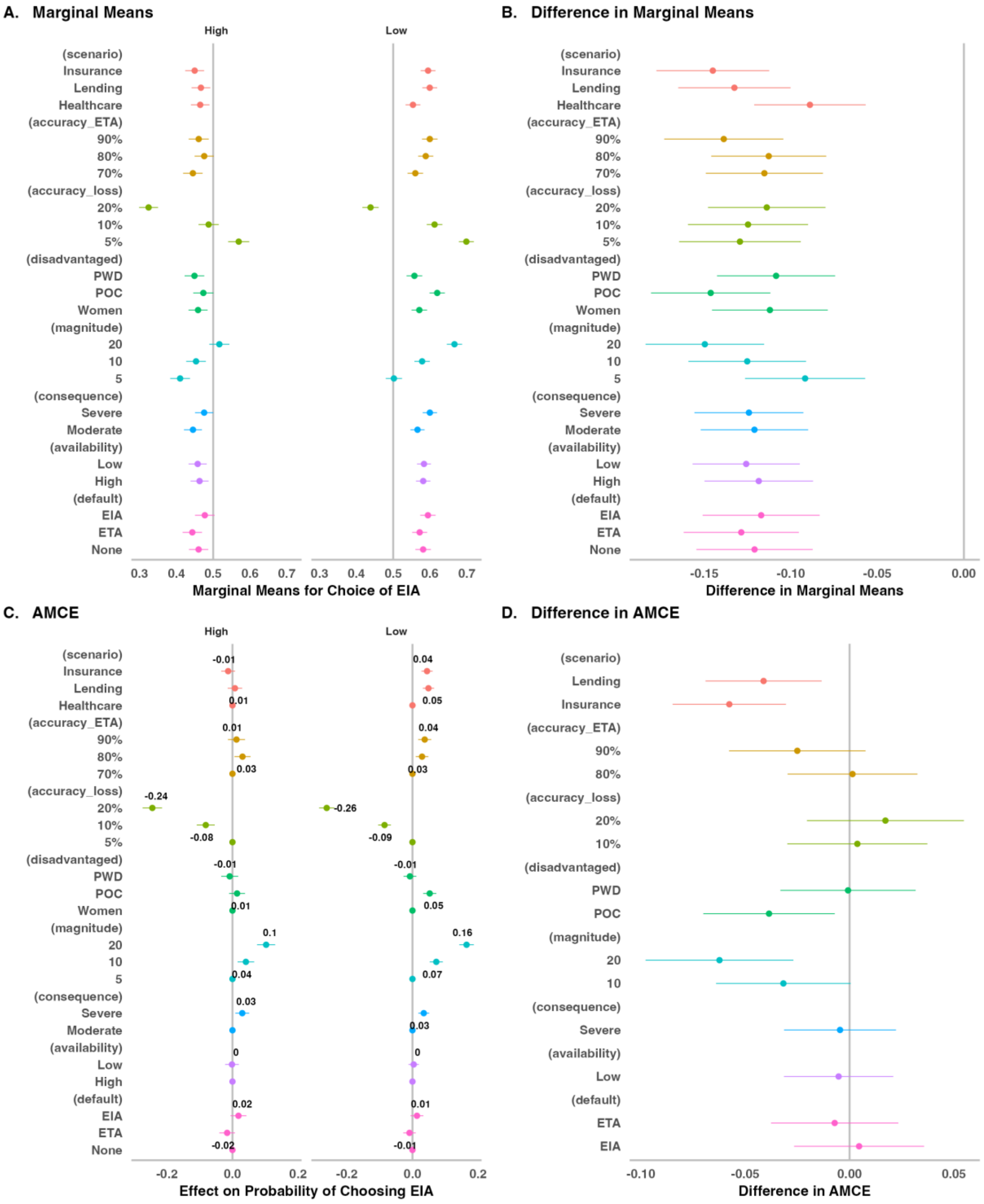

Note. Scores of 3 or lower on the entitlement dimension of the BJSO scale were coded as low entitlement orientation. Scores higher than 3 were coded as high entitlement orientation.

Table S22A. Marginal Means – Low Entitlement Orientation

Attribute	Level	Estimate	SE	Lower CI	Upper CI
Scenario	Healthcare	0.55	0.01	0.53	0.57
Scenario	Lending	0.60	0.01	0.58	0.62
Scenario	Insurance	0.59	0.01	0.57	0.61
ETA Accuracy	70%	0.56	0.01	0.54	0.58
ETA Accuracy	80%	0.59	0.01	0.57	0.61
ETA Accuracy	90%	0.60	0.01	0.58	0.62
Accuracy Loss	5%	0.70	0.01	0.68	0.72
Accuracy Loss	10%	0.61	0.01	0.59	0.63
Accuracy Loss	20%	0.44	0.01	0.42	0.46
Disadvantaged Group	Women	0.57	0.01	0.55	0.59
Disadvantaged Group	POC	0.62	0.01	0.60	0.64
Disadvantaged Group	PWD	0.56	0.01	0.54	0.58
Magnitude	5%	0.50	0.01	0.48	0.52
Magnitude	10%	0.58	0.01	0.56	0.60
Magnitude	20%	0.67	0.01	0.65	0.69
Consequence	Moderate	0.57	0.01	0.55	0.59
Consequence	Severe	0.60	0.01	0.58	0.62
Availability	High	0.58	0.01	0.56	0.60
Availability	Low	0.58	0.01	0.56	0.60
Default	None	0.58	0.01	0.56	0.60
Default	ETA	0.57	0.01	0.55	0.59
Default	EIA	0.59	0.01	0.57	0.62

Table S22B. Marginal Means – High Entitlement Orientation

Attribute	Level	Estimate	SE	Lower CI	Upper CI
Scenario	Healthcare	0.46	0.01	0.44	0.49
Scenario	Lending	0.47	0.01	0.44	0.49
Scenario	Insurance	0.45	0.01	0.42	0.48
ETA Accuracy	70%	0.44	0.01	0.42	0.47
ETA Accuracy	80%	0.48	0.01	0.45	0.50
ETA Accuracy	90%	0.46	0.01	0.43	0.49
Accuracy Loss	5%	0.57	0.01	0.54	0.60
Accuracy Loss	10%	0.49	0.01	0.46	0.52
Accuracy Loss	20%	0.32	0.01	0.30	0.35
Disadvantaged Group	Women	0.46	0.01	0.43	0.48
Disadvantaged Group	POC	0.47	0.01	0.45	0.50
Disadvantaged Group	PWD	0.45	0.01	0.42	0.48
Magnitude	5%	0.41	0.01	0.38	0.44
Magnitude	10%	0.45	0.01	0.43	0.48
Magnitude	20%	0.52	0.01	0.49	0.54
Consequence	Moderate	0.44	0.01	0.42	0.47
Consequence	Severe	0.48	0.01	0.45	0.50
Availability	High	0.46	0.01	0.44	0.49
Availability	Low	0.46	0.01	0.43	0.48
Default	None	0.46	0.01	0.43	0.49
Default	ETA	0.44	0.01	0.42	0.47
Default	EIA	0.48	0.01	0.45	0.50

Table S23. Difference in Marginal Means – High - Low Entitlement Orientation

**Attribute**	**Level**	**Estimate**	**SE**	***z***	***p***	**Lower CI**	**Upper CI**
Scenario	Healthcare	-0.09	0.02	-5.41	<0.01	-0.12	-0.06
Scenario	Lending	-0.13	0.02	-8.01	<0.01	-0.17	-0.10
Scenario	Insurance	-0.15	0.02	-8.73	<0.01	-0.18	-0.11
ETA Accuracy	70%	-0.12	0.02	-6.68	<0.01	-0.15	-0.08
ETA Accuracy	80%	-0.11	0.02	-6.65	<0.01	-0.15	-0.08
ETA Accuracy	90%	-0.14	0.02	-7.90	<0.01	-0.17	-0.10
Accuracy Loss	5%	-0.13	0.02	-7.22	<0.01	-0.16	-0.09
Accuracy Loss	10%	-0.12	0.02	-7.05	<0.01	-0.16	-0.09
Accuracy Loss	20%	-0.11	0.02	-6.58	<0.01	-0.15	-0.08
Disadvantaged Group	Women	-0.11	0.02	-6.57	<0.01	-0.15	-0.08
Disadvantaged Group	POC	-0.15	0.02	-8.32	<0.01	-0.18	-0.11
Disadvantaged Group	PWD	-0.11	0.02	-6.23	<0.01	-0.14	-0.07
Magnitude	5%	-0.09	0.02	-5.18	<0.01	-0.13	-0.06
Magnitude	10%	-0.13	0.02	-7.22	<0.01	-0.16	-0.09
Magnitude	20%	-0.15	0.02	-8.57	<0.01	-0.18	-0.12
Consequence	Moderate	-0.12	0.02	-7.64	<0.01	-0.15	-0.09
Consequence	Severe	-0.12	0.02	-7.73	<0.01	-0.16	-0.09
Availability	High	-0.12	0.02	-7.41	<0.01	-0.15	-0.09
Availability	Low	-0.13	0.02	-7.96	<0.01	-0.16	-0.09
Default	None	-0.12	0.02	-7.06	<0.01	-0.15	-0.09
Default	ETA	-0.13	0.02	-7.58	<0.01	-0.16	-0.10
Default	EIA	-0.12	0.02	-6.79	<0.01	-0.15	-0.08

Table S24A. AMCE – Low Entitlement Orientation

Attribute	Level	Estimate	SE	*z*	*p*	Lower CI	Upper CI
Scenario	Healthcare	0.00					
Scenario	Lending	0.05	0.01	5.59	<0.01	0.03	0.07
Scenario	Insurance	0.04	0.01	5.11	<0.01	0.03	0.06
ETA Accuracy	70%	0.00			<0.01		
ETA Accuracy	80%	0.03	0.01	2.98	<0.01	0.01	0.05
ETA Accuracy	90%	0.04	0.01	3.68	<0.01	0.02	0.06
Accuracy Loss	5%	0.00			<0.01		
Accuracy Loss	10%	-0.09	0.01	-8.61	<0.01	-0.10	-0.07
Accuracy Loss	20%	-0.26	0.01	-22.18	<0.01	-0.28	-0.24
Disadvantaged Group	Women	0.00			<0.01		
Disadvantaged Group	POC	0.05	0.01	5.14	<0.01	0.03	0.07
Disadvantaged Group	PWD	-0.01	0.01	-0.78	0.44	-0.03	0.01
Magnitude	5%	0.00					
Magnitude	10%	0.07	0.01	7.11	<0.01	0.05	0.09
Magnitude	20%	0.16	0.01	14.60	<0.01	0.14	0.19
Consequence	Moderate	0.00			<0.01		
Consequence	Severe	0.03	0.01	4.08	<0.01	0.02	0.05
Availability	High	0.00					
Availability	Low	0.00	0.01	0.46	0.64	-0.01	0.02
Default	None	0.00					
Default	ETA	-0.01	0.01	-0.94	0.35	-0.03	0.01
Default	EIA	0.01	0.01	1.37	0.17	-0.01	0.03

Table S24B. AMCE – High Entitlement Orientation

Attribute	Level	Estimate	SE	*z*	*p*	Lower CI	Upper CI
Scenario	Healthcare	0.00					
Scenario	Lending	0.01	0.01	0.67	0.50	-0.01	0.03
Scenario	Insurance	-0.01	0.01	-1.23	0.22	-0.03	0.01
ETA Accuracy	70%	0.00					
ETA Accuracy	80%	0.03	0.01	2.45	0.01	0.01	0.06
ETA Accuracy	90%	0.01	0.01	0.92	0.36	-0.01	0.04
Accuracy Loss	5%	0.00					
Accuracy Loss	10%	-0.08	0.01	-5.83	<0.01	-0.11	-0.05
Accuracy Loss	20%	-0.24	0.02	-16.14	<0.01	-0.27	-0.21
Disadvantaged Group	Women	0.00					
Disadvantaged Group	POC	0.01	0.01	1.13	0.26	-0.01	0.04
Disadvantaged Group	PWD	-0.01	0.01	-0.64	0.52	-0.03	0.02
Magnitude	5%	0.00					
Magnitude	10%	0.04	0.01	3.17	<0.01	0.02	0.07
Magnitude	20%	0.10	0.01	7.27	<0.01	0.07	0.13
Consequence	Moderate	0.00					
Consequence	Severe	0.03	0.01	2.77	0.01	0.01	0.05
Availability	High	0.00					
Availability	Low	0.00	0.01	-0.14	0.89	-0.02	0.02
Default	None	0.00					
Default	ETA	-0.02	0.01	-1.33	0.18	-0.04	0.01
Default	EIA	0.02	0.01	1.47	0.14	-0.01	0.04

Table S25. Difference in AMCE – High - Low Entitlement Orientation

**Attribute**	**Level**	**Estimate**	**SE**	***z***	***p***	**Lower CI**	**Upper CI**
Scenario	Lending	-0.04	0.01	-2.90	<0.01	-0.07	-0.01
Scenario	Insurance	-0.06	0.01	-4.16	<0.01	-0.08	-0.03
ETA Accuracy	80%	0.00	0.02	0.09	0.93	-0.03	0.03
ETA Accuracy	90%	-0.03	0.02	-1.50	0.13	-0.06	0.01
Accuracy Loss	10%	0.00	0.02	0.22	0.83	-0.03	0.04
Accuracy Loss	20%	0.02	0.02	0.90	0.37	-0.02	0.05
Disadvantaged Group	POC	-0.04	0.02	-2.40	0.02	-0.07	-0.01
Disadvantaged Group	PWD	0.00	0.02	-0.04	0.96	-0.03	0.03
Magnitude	10%	-0.03	0.02	-1.93	0.05	-0.06	0.00
Magnitude	20%	-0.06	0.02	-3.45	<0.01	-0.10	-0.03
Consequence	Severe	0.00	0.01	-0.33	0.74	-0.03	0.02
Availability	Low	-0.01	0.01	-0.39	0.69	-0.03	0.02
Default	ETA	-0.01	0.02	-0.46	0.65	-0.04	0.02
Default	EIA	0.00	0.02	0.29	0.77	-0.03	0.04

Table S26. Fixed effects for the choice of EIA – Study 2 full data

Term	b (SE)	OR [95% CI]	p-value
(Intercept)	-0.25 (0.11)	0.78 [0.63, 0.97]	.025
Accuracy Loss [5% vs. 2%]	-0.75 (0.17)	0.47 [0.34, 0.66]	< .001
Accuracy Loss [10% vs. 5%]	-1.18 (0.16)	0.31 [0.22, 0.43]	< .001
Accuracy Loss [15% vs. 10%]	-1.19 (0.17)	0.30 [0.22, 0.43]	< .001
Accuracy Loss [20% vs. 15%]	0.19 (0.17)	1.21 [0.86, 1.70]	0.264
Impact Disparity [10% vs. 4%]	0.44 (0.27)	1.55 [0.91, 2.63]	0.107
Impact Disparity [20% vs. 10%]	0.96 (0.28)	2.62 [1.53, 4.48]	< .001

Figure S5. Effects of accuracy loss and impact disparity on choice of the EIA – Study 2 full data

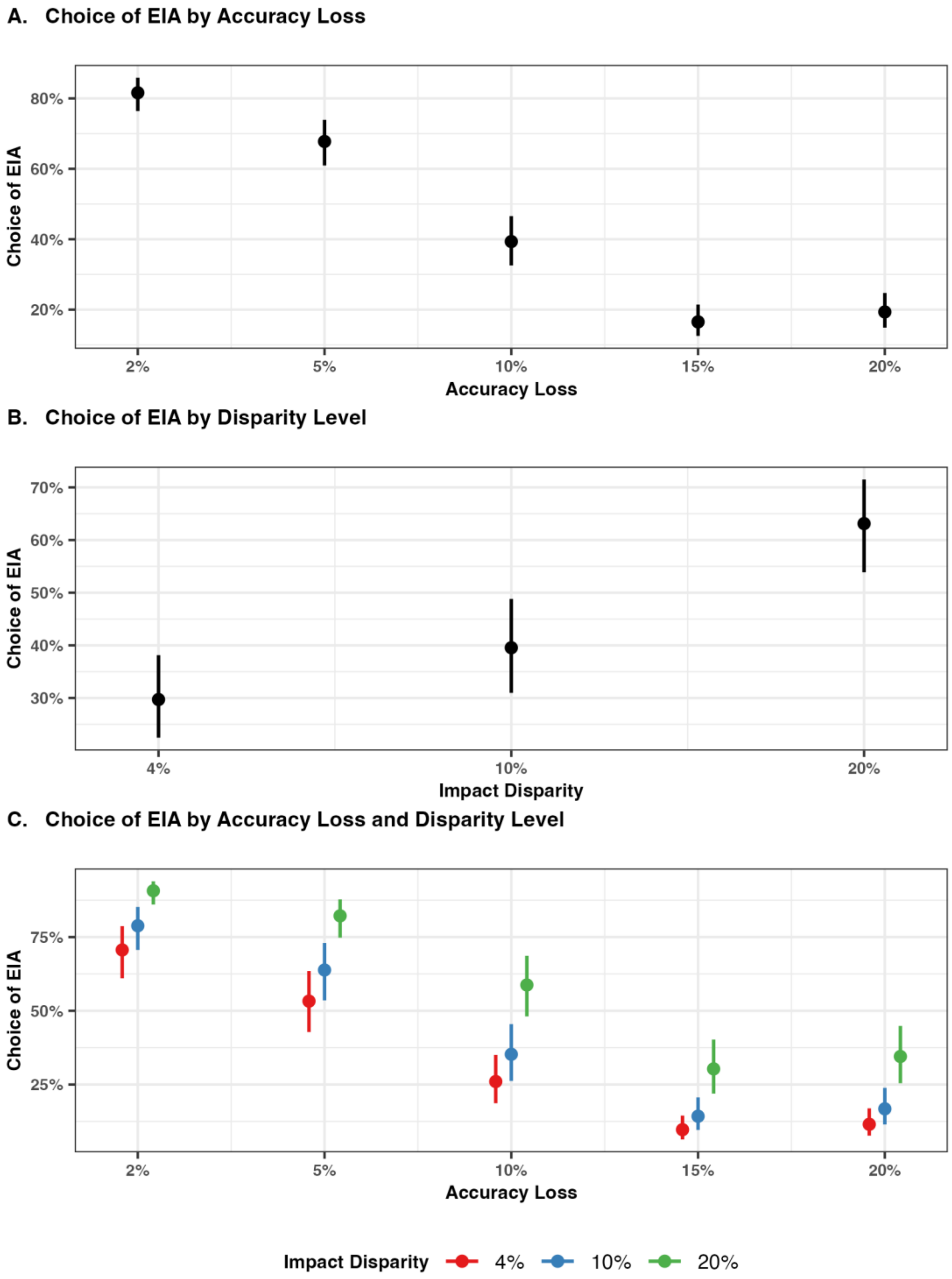

Figure S6. Probability of Choosing the EIA by Individual Characteristics – Study 2

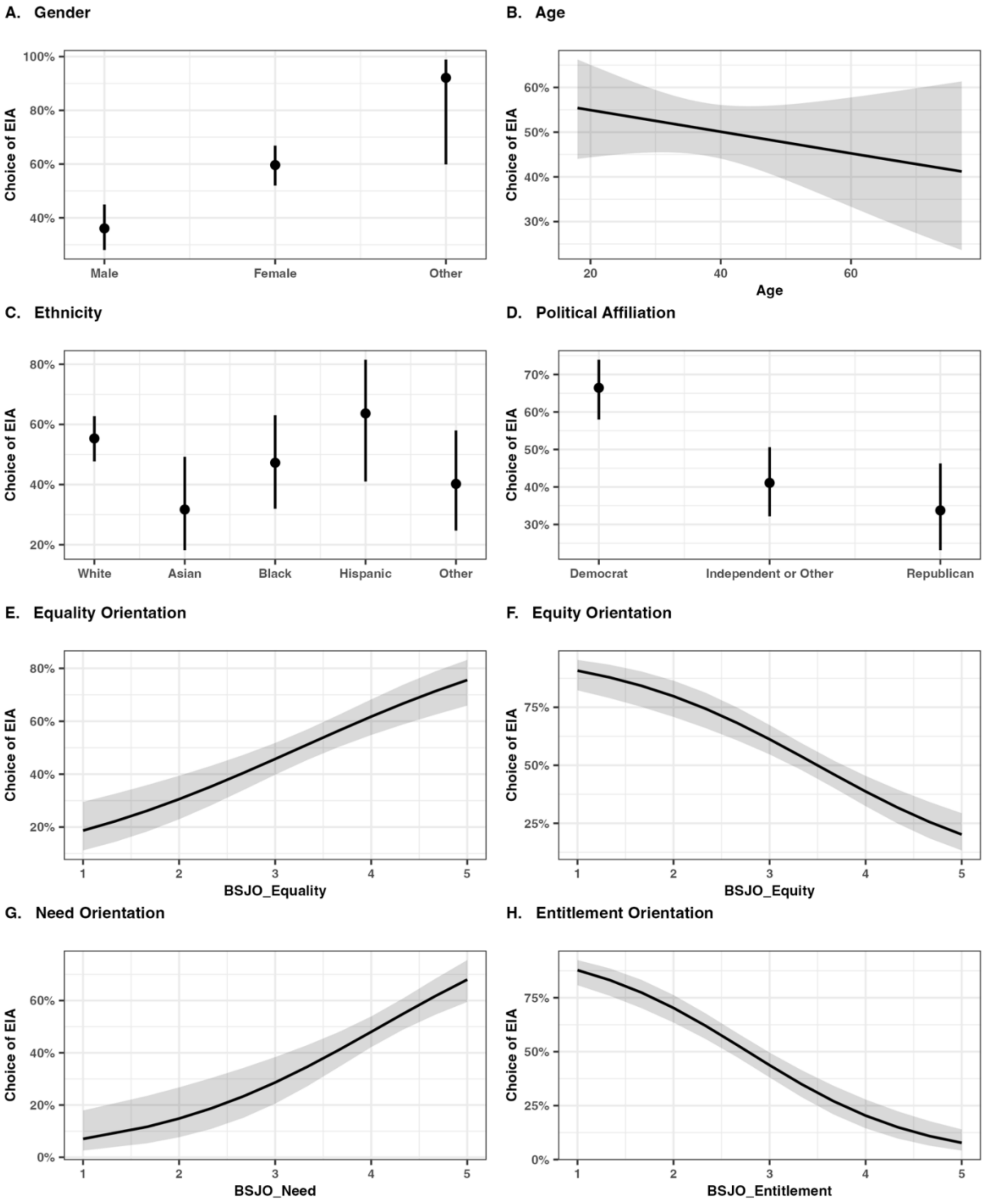

Supplement: S1 File — (PDF) [file pone.0319861.s001.pdf]
